# Switches, Excitable Responses and Oscillations in the Ring1B/Bmi1 Ubiquitination System

**DOI:** 10.1371/journal.pcbi.1002317

**Published:** 2011-12-15

**Authors:** Lan K. Nguyen, Javier Muñoz-García, Helene Maccario, Aaron Ciechanover, Walter Kolch, Boris N. Kholodenko

**Affiliations:** 1 Systems Biology Ireland, University College Dublin, Belfield, Dublin, Ireland; 2 Cancer and Vascular Biology Research Center, The Rappaport Faculty of Medicine and Research Institute, Technion-Israel Institute of Technology, Bat Galim, Haifa, Israel; 3 Department of Pathology, Anatomy and Cell Biology, Thomas Jefferson University, Philadelphia, Pennsylvania, United States of America; University of Illinois at Urbana-Champaign, United States of America

## Abstract

In an active, self-ubiquitinated state, the Ring1B ligase monoubiquitinates histone H2A playing a critical role in Polycomb-mediated gene silencing. Following ubiquitination by external ligases, Ring1B is targeted for proteosomal degradation. Using biochemical data and computational modeling, we show that the Ring1B ligase can exhibit abrupt switches, overshoot transitions and self-perpetuating oscillations between its distinct ubiquitination and activity states. These different Ring1B states display canonical or multiply branched, atypical polyubiquitin chains and involve association with the Polycomb-group protein Bmi1. Bistable switches and oscillations may lead to all-or-none histone H2A monoubiquitination rates and result in discrete periods of gene (in)activity. Switches, overshoots and oscillations in Ring1B catalytic activity and proteosomal degradation are controlled by the abundances of Bmi1 and Ring1B, and the activities and abundances of external ligases and deubiquitinases, such as E6-AP and USP7.

## Author Summary

The generation of polyubiquitin chains on target proteins as a degradation signal was a landmark discovery rewarded by the 2004 Nobel Prize in Chemistry. However, emerging evidence suggests that protein ubiquitination is more versatile. Different types of ubiquitin chains serve numerous non-proteolytic functions, among them regulation of the biological activities of target proteins. Here we demonstrate a flexible role of ubiquitination in the dynamic control of Ring1B, a ubiquitin ligase that monoubiquitinates histone H2A, which in turn silences gene expression. Remarkably, Ring1B increases its own activity by self-ubiquitination. A binding partner of Ring1B, Bmi1, facilitates Ring1B self-ubiquitination and protects both proteins from rapid degradation. We use computational modeling to show that the Ring1B/Bmi1 system can act as analog-digital converter, generating abrupt switches, multistable dynamics, oscillations and excitable overshoots. For instance, an increase in Bmi1 abundance brings about an abrupt “On” switch of Ring1B monoubiquitinating activity and downregulation of H2A-controlled genes, while a decrease in Bmi1 leads to an “Off” switch. These digital responses can display hysteresis, creating the biological memory. Distinct types of Ring1B activity responses (oscillatory, bistable and excitable) facilitate signal discrimination and allow the Ring1B/Bmi1/H2A system to distinctly affect gene silencing and potentially trigger different cell fates.

## Introduction

Recent discoveries have revolutionized our perception of the role of protein ubiquitination in signaling networks. Although initially ubiquitination was considered as a signal for proteasomal degradation, emerging evidence suggests that different types of ubiquitin chains may have non-proteolytic roles and can dramatically alter the biological activities of a target protein [1]. Early work showed that a polyubiquitin chain consisting of at least four ubiquitin molecules, which are linked through Lys^48^ (K48) initiates the rapid degradation of a target protein by the ubiquitin-proteasome system (UPS) [2]. Later, non-degradative roles of K63-linked oligo- and polyubiquitin chains were found for proteins involved in the DNA-damage response, the JNK, p38 MAPK and NF-κB signaling pathways, and endocytic trafficking [1]. Recently, atypical, branched ubiquitin chains that involve K6/K27/K48 ubiquitin linkages were discovered on the E3 ligase Ring1B that monoubiquitinates histone H2A. Interestingly, these atypical ubiquitin chains were generated only by Ring1B self-ubiquitination (also referred to as auto-ubiquitination), whereas Ring1B ubiquitination by the E3 ligase E6-AP (E6-associated protein) resulted in canonical K48 linkages [3].

The E3 ligase Ring1B is a RING finger protein, which interacts with another RING finger protein, Bmi1. Together with Polyhomeotic 1 and Chromobox protein homologue 4, Ring1B and Bmi1 form the core human Polycomb transcriptional Repressive Complex 1 (PRC1), which plays a critical regulatory role in the control of genes during development, ageing and cancer [4], [5], [6]. Owing to Ring1B catalytic activity, PRC1 is a major E3 ligase of histone H2A in vivo. Monoubiquitinated H2A (uH2A) represses transcriptional initiation and elongation [7], [8], [9], leading to gene silencing that was implicated in tumorigenesis and stem cell development [4], [6], [10], [11]. Increased monoubiquitination of H2A was observed upon UV radiation in mammalian cells, implying a role of uH2A in the DNA damage response and/or DNA repair-induced chromatin remodelling [12], [13]. By contrast, uH2A deubiquitination was found to facilitate cell cycle progression where repressive histone marks are removed during G0-G1/S transition to allow S-phase gene expression [14], [15]. Thus, Ring1B-induced H2A monoubiquitination (and subsequent deubiquitination) plays an essential role in regulating gene expression.

Both Ring1B and Bmi1 are short-lived proteins, which are degraded by UPS. It has long been understood that self-ubiquitination of RING finger-containing E3 ligases targets them for UPS-mediated destruction [16], [17]. Surprisingly, recent work reveals that degradation of Ring1B is independent of its self-ubiquitinating activity [18]. Self-ubiquitination of Ring1B generates branched K6/K27 ubiquitin chains, and this is required for efficient *in vitro* monoubiquitination of histone H2A, whereas canonical K48-linked chains, generated by other ligases target Ring1B for degradation. The presence of Bmi1 greatly facilitates Ring1B monoubiquitinating activity with respect to H2A, and the association between Ring1B and Bmi1 protects these proteins from rapid degradation [18].

Similar to protein phosphorylation/dephosphorylation, ubiquitination is reversed by the opposing process of deubiquitination. Ubiquitination chains with distinct linkages and structures are recognized and deubiquitinated by different deubiquitinases (DUBs) that feature specialized ubiquitin-binding domains. Studies of protein phosphorylation have shown that upon small changes in input kinase or phosphatase activities, target proteins can abruptly switch between distinct phosphorylation states, a phenomenon termed “ultrasensititvity” [19]. Moreover, phosphorylation on two or more residues not only increases ultrasensitivity, but potentially leads to bistability or multistability where under the same input conditions, a target protein can reside in any of two or more stable stationary states with different phosphorylation levels [20], [21]. Recently, it has been shown that *intermolecular* auto-phosphorylation, a salient feature of activation of many protein kinases [22], [23], [24] can bring about the intricate dynamic behavior that involves abrupt activity switches, bistability and hysteresis [25]. Interestingly, when the phosphorylation dynamics are bistable, multiplicity of deactivation routes can result in sustained, pulsatory oscillations in kinase activities [25].

Ubiquitination reaction circuitry is more complex than (de)phosphorylation cycles. Two enzymes, ubiquitin-activating (E1) and ubiquitin-conjugating (E2) enzymes, are involved in every ubiquitin molecule transfer to a target protein by ligase E3, and ubiquitin molecules can form polymeric chains of different structures, which is not the case for phosphorylation. Yet, there is a striking similarity between intermolecular auto-phosphorylation of protein kinases and auto-ubiquitination that results in self-activation or self-inhibition of E3 ligases. For instance, auto-ubiquitination of Itch ligase was shown to be an *intermolecular* reaction, which generated K63-linkages, rather than the K48-linked chains that target Itch for proteasomal degradation [26]. Likewise, self-ubiquitination of the HECT-type E3 ligase Nedd4 leads to better recognition and higher rate of monoubiquitination of Eps15 by Nedd4 in the EGFR internalisation and degradation pathway [27], whereas auto-ubiquitination of DIAP1(*Drosophila* inhibitor of apoptosis protein 1), an E3 ligase responsible for cell death regulation in *Drosophila*, serves to attenuate DIAP1 ligase activity towards its substrates (such as the proapoptotic protein Rpr) via formation of K63-linkages rather than K48-based polyubiquitin chains [28]. *In vitro* data suggest that two Ring1B molecules can self-dimerize via their C-terminal domains [29], reiterating the possibility of intermolecular auto-ubiquitination.

The present paper shows that extremely complex Ring1B activity and degradation dynamics (that results in the intricate temporal control of H2A monoubiquitination) can be brought about by the Ring1B - Bmi1 interaction circuitry, intermolecular Ring1B auto-ubiquitination, and Ring1B (de)ubiquitination by external ligases and DUBs. Using computational modelling to elucidate these dynamics, we demonstrate that the Ring1B/Bmi1/H2A network can display oscillatory, bistable and excitable behaviors. We show that overexpression (or mutation) of Bmi1 and a deubiquitinating enzyme USP7 do not merely change the amplitude of Ring1B degradation and catalytic rates, but dramatically transform their response dynamics. For instance, an increase in Bmi1 abundance can bring about bistable, all-or-none Ring1B monoubiquitination activity and bistable, all-or-none expression of H2A-controlled genes. Under the proper conditions, which include the upregulation of USP7 and Bmi1, self-perpertuating oscillatory responses of Ring1B monoubiquitination are facilitated. In the proximity of oscillatory regimes, the Ring1B/Bmi1 system displays an excitable behavior where a transient perturbation causes Ring1B activity and degradation rate to overshoot before returning to the basal level. Our findings unveil the intrinsic complexity of the dynamics of Ring1B activity and H2A monoubiquitination and allow for direct experimental testing.

## Model

### Key Experimental Observations and Kinetic Model Building

**Figure 1:**
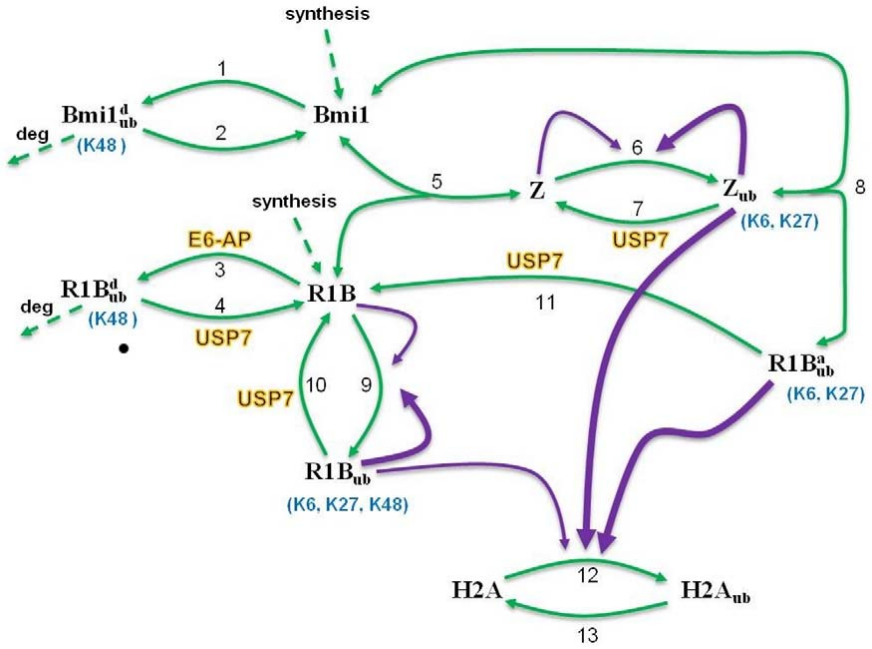
Kinectic scheme of the core Ring1B/Bmi1 ubiquitination system. This scheme was used to build the mathematical models; the reactions are described in the text. Protein-protein interactions and (de)ubiquitination reactions are shown by solid green lines. Reactions of proteosomal degradation and *de novo* synthesis of Bmi1 and Ring1B (shown by dashed lines) are neglected on short-timescales (<1 hr). Catalytic intermolecular interactions are shown by purple lines (the arrow thickness indicates levels of catalytic activity). R1B is Ring1B; Bmi1^d^_ub_ and R1B^d^_ub_ are ubiquitinated forms of Bmi1 and Ring1B targeted for degradation; Z is the complex of Bmi1 and Ring1B; R1B_ub_, R1B^a^_ub_ and Z_ub_ are self-ubiquitinated forms of Ring1B (free or associated with Bmi1, see text for details).

We developed a computational model that encapsulates key molecular interactions and reveals intricate dynamic behaviors of the Ring1B/Bmi1/H2A network. The model is based on a careful examination of all available biological data and accounts for distinct, Bmi1-dependent and independent modes of Ring1B self-induced ubiquitination, Ring1B and Bmi1 ubiquitination by external ligases and monoubiquitination of histone H2A by catalytically active forms of Ring1B (Fig. 1). We modelled the system on two different timescales: (i) a short timescale (<1 hour) where degradation reactions occur but can be neglected, as the protein abundances in the system have not yet changed much; and (ii) a long timescale (>1 hour), where protein synthesis and degradation are explicitly considered in the model. We then show that the inclusion of protein synthesis and degradation rates does not practically change the network dynamics observed on short timescales, where the key biochemical modifications take place that precede and in part determine changes in protein degradation.

Below, we describe underlying core biochemical mechanisms and key biological observations which led to the assumptions built into the model.

#### Ubiquitination of Ring 1B and Bmi1 by other ubiquitin ligases

Ring1B is targeted for degradation by the “external” E6-AP ligase that generates “canonical” K48-linkages [3], which are different from the atypical, mixed K6, K27 and K48-linkages resulting from Ring1B self-ubiquitination. Bmi1 that interacts with Ring1B has no self-ubiquitinating activity and is ubiquitinated by another external ligase consisting of Speckle-type POZ protein (SPOP) and Cullin 3(Cul-3) [30]. In the kinetic diagram in Fig. 1, reactions 1 and 3 describe the ubiquitination of Bmi1 and Ring1B by these external E3 ligases, yielding Bmi1^d^_ub_ and R1B^d^_ub_, respectively. The Bmi1^d^_ub_ and R1B^d^_ub_ forms mainly contain K48-linkages and are subsequently degraded by UPS, as shown by dashed lines in Fig. 1. Bmi1^d^_ub_ and R1B^d^_ub_ also can be deubiquitinated by deubiquitinases (DUBs) and converted back to their un-ubiquitinated forms in reactions 2 and 4.

#### Bmi1-Ring1B complex formation protects both proteins from degradation

Ring1B and Bmi1 form a heterodimeric complex (Z, the product of reversible reaction 5, Fig. 1) and stabilize each other through direct interactions against degradation. This increases the half-life of Ring1B and Bmi1 up to three fold [18]. In the complex Z, the RING domain of Bmi1 is embraced by the N-terminal arm of Ring1B, although mutual stabilization is independent of Ring1B ligase activity [4], [18], [31].

#### Distinct modes of self-ubiquitination and activation of Ring1B

Self-ubiquitination of Ring1B occurs through two different reaction routes. Free Ring1B is the substrate of the first autocatalytic, self-ubiquitination reaction (reaction 9, producing R1B_ub_, Fig. 1), whereas in the second autocatalytic reaction, Ring1B is associated with Bmi1 (reaction 6 yielding complex Z_ub_, Fig. 1). Importantly, the E6-AP ligase modifies the same lysine residues on Ring1B, which are modified in two self-ubiquitination reactions. This makes all three modes of ubiquitination mutually exclusive [3], and they are shown as competitive reactions in Fig. 1. We assume that (i) both autocatalytic reactions 6 and 9 occur through intermolecular rather than intramolecular interactions, and (ii) a self-ubiquitinated Ring1B molecule can ubiquitinate another Ring1B molecule [29].

Experiments with two sets of ubiquitin mutants (where all lysine residues except one or only a single lysine residue were substituted with arginine) show that two self-ubiquitination reactions 9 and 6 generate distinct ubiquitin chains on Ring1B [18]. Auto-ubiquitination of free Ring1B (reaction 9) creates atypical, mixed K6, K27 and K48 linkages on R1B_ub_, whereas self-ubiquitinated Ring1B in complex Z_ub_ (reaction 6) does not contain K48 linkages, and ubiquitin conjugates associated with Z_ub_ have lower molecular-mass. Most importantly, *in vitro* data suggest that distinct polyubiquitin chains endow Ring1B with different catalytic activities with respect to H2A; Z_ub_ showed much stronger monoubiquititation activity than R1B_ub_ [18]. In the reversible reaction 8 Z_ub_ dissociates yielding Bmi1 and R1B^a^_ub_, another ubiquitinated form of Ring1B which is distinct from R1B_ub_ (Fig. 1). Similarly to Z_ub_, R1B^a^_ub_ contains primarily K6 and K27 linkages rather than K48 linkages.

The ubiquitinated forms, Z_ub_, R1B_ub_, and R1B^a^_ub_ are deubiquitinated by DUBs (reactions 4, 7, 10 and 11, Fig. 1). USP7 was recently discovered as a DUB that does not discriminate between the activating and proteolysis-targeting modes of Ring1B ubiquitination [32], and thus can catalyse all deubiquitination reactions.

#### H2A monoubiquitination by active Ring1B

H2A is monoubiquitinated into H2A_ub_, which is shown as reaction 12 in Fig. 1. We assume that all three forms of Ring1B (R1B_ub_, R1B^a^_ub_ and Z_ub_) act as ligases that monoubiquitinate H2A, and their catalytic activities are ranked in decreasing order, Z_ub_ > R1B^a^_ub_ >> R1B_ub_ [18]. H2A_ub_ is deubiquitinated in reaction 13.

#### Kinetic equations

The rate equations and parameter values can be found in the Supporting Information (SI), Eqns S1, S2, S3, S4, S5, S6, S7, S8, S9, S10 and Table S1. The lack of experimentally measured kinetic data remains a challenge for computational modeling. For both the short and long timescale models, the kinetic parameters have been constrained by experimental data (wherever available) or typical values for protein association/dissociation and enzymatic reaction rates. The rate constants of Ring1B and Bmi1 degradation for our long timescale model are derived from the experimental data on the half-lives of free Ring1B and Bmi1 [18]. The rates of protein-protein interactions are given by the mass-action (MA) law, and the rates of reactions catalyzed by “external” E3 ligases and DUBs are described by the Michaelis-Menten (MM) expressions, which are often exploited to describe the rates of protein modification reactions, for instance those catalyzed by kinases and phosphatases [33], [34], [35]. When the MM constant of a particular ligase or DUB-catalyzed reaction is substantially larger than the concentration of the corresponding substrate (e.g., a Ring1B form or the total Ring1B abundance), the concentration of the enzyme-substrate complex can be neglected and the reaction rate is approximated by a linear expression (used for some reactions for simplicity, see Table S1).

A MM kinetic description is an approximation, which reduces the number of reactions analyzed in the model by assuming that the enzyme-substrate complexes can be considered at quasi-steady state (QSS) conditions. For metabolic pathways, this approximation is commonly accepted because the enzyme concentrations are usually much lower than the substrate concentrations, but for signaling and gene networks, the applicability of the QSS approximation and the MM kinetics requires further analysis, as the enzyme and substrate concentrations are often comparable [36], [37]. A more precise total QSS approximation considers explicitly the concentrations of enzyme-substrate complexes, but also assumes that these complexes can be considered at quasi-equilibrium [38], [39]. A description of a signaling process at the elementary step level (where reactions follow the MA kinetics) clearly circumvents the question of the applicability of a QSS model reduction (where these elementary steps are lumped into a single MM reaction). However in some cases, a reduced MM description and a precise MA description at the elementary step level may lead to different dynamic behaviors exhibited by the corresponding models [21], [37], [40]. Therefore, we have made extensive simulations to prove that the intricate Ring1B/Bmi1/H2A dynamics demonstrated here for an approximate MM kinetic description holds true for a precise mass-action description at the elementary step level (see below and supplementary Text S1). Importantly, for two different scenarios when the MM approximation is (*i*) justified or (*ii*) inapplicable, we observed that the complex dynamics (bistability, oscillations and excitability) persist over a wide range of parameter values for both detailed and reduced models, suggesting that these dynamics are a robust systems property.

## Results

### Digital “On” or “Off” Monoubiquitination of Histone H2A Arises from Ring1B - Bmi1 Autocatalytic Activation

A commonly observed behavior of protein ubiquitination is a gradual approach to a (quasi-) stationary state featuring specific ubiquitination levels of targets. This behavior can also be observed for the Ring1B/Bmi1/H2A network that displays single stable steady states for a wide array of kinetic parameter values. For instance, when the Ring1B/Bmi1 ubiquitination assays have been carried out in a cell-free reconstituted system, the system quickly relaxes to (quasi-) steady state conditions [18]. Yet, in certain parameter ranges, the Ring1B system exhibits intricate dynamic behavior that may be exploited by cells to efficiently control H2A monoubiquitination and gene expression.

Although the characteristic timescales of the (de)activation of the Ring1B/Bmi1 system in live cells have not been well documented, the available data suggest that the post-translational, (de)ubiquitination dynamics is much faster than the rates of protein synthesis and degradation. (De)ubiquitination typically occurs on the timescale of seconds to minutes [41], [42], whereas Ring1B/Bmi1 degradation and *in vivo* synthesis evolve on the timescale of several hours [18]. Yet, it is still unknown what the specific signaling inputs to the Ring1B/Bmi1/H2A system *in vivo* are, and whether these inputs are transient or sustained. Therefore, our model largely focuses on the post-translational modification dynamics of the Ring1B/Bmi1/H2A system on the relatively short timescale within an hour after abrupt changes in the input. As we will see below, these input changes can result in digital “on” or “off” H2A monoubiquitination responses. We subsequently examine the Ring1B/Bmi1/H2A dynamics on the long timescale taking into account protein synthesis and degradation and show that during the first hour, a long timescale model behaves practically indistinguishable from a short-scale model that neglects protein synthesis and degradation.

#### Prerequisites of bistable behavior

Bistability is a phenomenon in which a dynamic system switches between two distinct stable steady states (often “On” and “Off” in activity terms), but cannot rest in an intermediate state. Likewise, multistability enables a system to switch between multiple stable states, thereby displaying digital responses to external cues [20]. Bistable and multistable behaviors can arise due to explicit or implicit positive feedback motifs [21], [43], [44] and are observed for many biological systems, such as involved in cell-fate determinations and cell cycle control [20], [45], [46], [47]. We show next that abrupt switches and bi- or multistability of Ring1B activity arise from intermolecular autoctalytic ubiquitination coupled with saturability of Ring1B deubiquitinating reactions.

**Figure 2:**
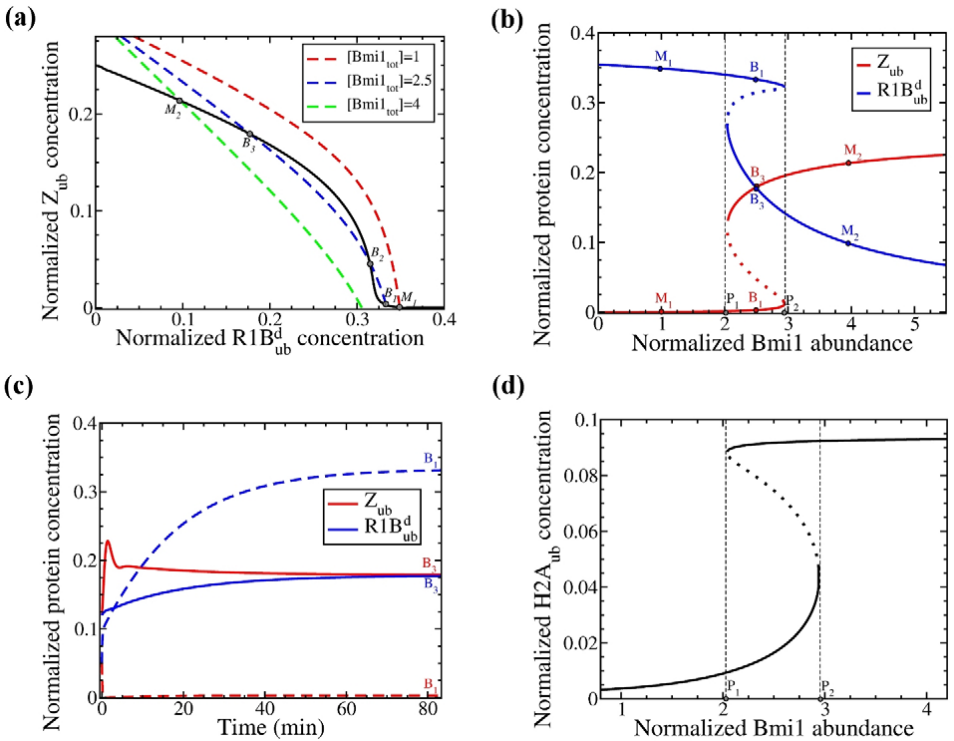
Bistability and hysteresis in the Ring1B/Bmi1 system. (**a**) Intersections between the QSS curves, 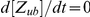 (black line) and 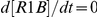 (dashed lines), determine the system steady states, which are shown on the [R1B^d^_ub_]-[Z_ub_] plane for different Bmi1 abundances (Bmi1_tot_). All concentrations are normalized by the Ring1B abundance (100 nM) and hereafter shown in dimensionless units. M_1_ and M_2_ are single, stable steady states (Bmi1_tot_ = 1 and 4, respectively). For Bmi1_tot_ = 2.5 three steady states exist; B_1_ and B_3_ are stable states, and B_2_ is an unstable state. (**b**) Dependence of the steady state levels of Z_ub_ (catalytically active form, red curve) and R1B^d^_ub_ (targeted for degradation form, blue) on the Bmi1 abundance. Unstable states are shown by dotted line. Designations of steady states are the same as in panel (**a**). Turning points P_1_ and P_2_ indicate saddle-node bifurcations. (**c**) Temporal dynamics of [Z_ub_] (red) and [R1B^d^_ub_] (blue) approaching steady states in the bistable region for two different initial conditions. Note that on this timescale, the behavior of two systems (where protein synthesis and degradation are neglected or considered) remains the same. For the initial high level of Bmi1^d^_ub_ ([Bmi1^d^_ub_] = 2.45, [Bmi1] = 0, [R1B^d^_ub_ ] = 0.05, [R1B] = 0.9, [Z] = 0, [Z _ub_] = 0.05, [R1B^a^_ub_] = 0, and [R1B_ub_] = 0), the system approaches a steady state with low Z_ub_ and high R1B^d^_ub_ concentrations (dashed lines). This steady state is indicated as B_1_ in panels (a) and (b). For the initial high levels of free Bmi ([Bmi1^d^_ub_] = 1.08, [Bmi1] = 1.1, [R1B^d^_ub_ ] = 0.12, [R1B] = 0.1, [Z] = 0.2, [Z _ub_] = 0.12, [R1B^a^_ub_] = 0.44, and [R1B_ub_] = 0.02), the system approaches a steady state with high Z_ub_ and low R1B^d^_ub_ concentrations (solid lines). This steady state is indicated B_3_ in panels (a) and (b). (**d**) Dependence of the stationary level of monoubiquitinated histone H2A on the Bmi1 abundance. The remaining parameter values are given in Table S1.

Autocatalytic activation of Ring1B by self-ubiquitination generates positive feedback loops where active Ring1B forms promote their own production (reactions 6 and 9, Fig. 1). This positive feedback is crucial for the emergence of bistable behavior. The occurrence of bistability can be illustrated by plotting quasi steady-state (QSS) curves for two variables (for instance, two Ring1B forms, R1B^d^_ub_ and Z_ub_) on one plane where steady states of the entire system correspond to intersection points. Fig. 2a illustrates that there can be one or three intersection points, depending on the total Bmi1 abundance, which shifts the QSS R1B^d^_ub_ curve (dashed lines) downward or upward on the plane, but does not change the QSS dependence of Z_ub_ (solid line). Single intersection points present monostable steady states, whereas the Bmi1 abundance range leading to three intersection points corresponds to bistability (see the stability analysis in Text S1). Importantly, these calculations show that saturability of Z_ub_ deubiquitination (reaction 7, see supplementary Table S1) is a second prerequisite of bistability given autocatalytic positive feedback. The assumption of reaction 7 saturability can be replaced by saturability of reaction 10. Below, for simplicity deubiquitination reactions other than reaction 7 are assumed to follow the apparent first-order kinetics. Note that bistability persists when the MM approximation, which is used in the reduced model, is replaced by a MA description that is exploited by a more accurate model at the elementary step level (see section 5, Text S1, Table S2, and Figs. S9 and S10).

#### Regulation of Ring1B catalytic and degradation rates by Bmi1 abundance

The model predicts that outside the bistability domain, the R1B^d^_ub_ fraction targeted for degradation is high at low Bmi1 abundance (as illustrated by point M_1_, Fig. 2b) and low when Bmi1 abundance is high (point M_2_), which agrees with experimental observations [18]. Temporal dynamics of R1B^d^_ub_ corresponding to these monostable regions are shown in Fig. S1. Importantly, when the Bmi1 abundance increases from low to high values, the level of the Bmi1-independent, self-ubiquitinated form, R1B_ub_, switches from high to low amplitudes, whereas the Bmi1-mediated, self-ubiquitinated forms, Z_ub_ and R1B^a^_ub_, exhibit opposite transitions from low to high levels (Fig. 2b and Fig. S2). Since R1B^a^_ub_ and Z_ub_ have greater monoubiquitinating activity towards H2A than R1B_ub_, the high Bmi1 abundance increases H2A monoubiquitination, whereas low Bmi1 abundance decreases it. This is in line with experimental observations that Bmi1 attenuates ubiquitination of free Ring1B and increases H2A monoubiquitination [18].

Inside the bistability domain, at the same Bmi1 abundance, the Ring1B catalytic and degradation rates can be very different at two distinct stable states. The state, in which the system resides, is determined by the previous history, which is the starting level of different Bmi1 and Ring1B forms. This phenomenon is illustrated by the temporal evolution of Z_ub_ and R1B^d^_ub_ in Fig. 2c. Given a fixed total Bmi1 level, if the system starts with a low level of free Bmi1 and a high level of Bmi1_ub_, catalytically active Z_ub_ (red) and R1B^d^_ub_ (blue) approach their low and high steady-state level, thereby Ring1B is targeted for degradation, whereas high initial Bmi1 levels result in low steady-state R1B^d^_ub_ and high steady-state Z_ub_ levels (dashed, blue and red lines, Fig. 2c).

Bistable systems can translate graded input changes into switch-like (digital) output responses and display memory, termed hysteresis, which means that the input must exceed a threshold to switch the system to another steady state, at which the system can remain, when the input decreases. Such properties are observed in the Ring1B/Bmi1 system where an incremental increase in the Bmi1 abundance starting from point M_1_ retains R1B^d^_ub_ at high level, but a further increase in Bmi1 abundance beyond a threshold level (P_2_, Fig. 2b) abruptly switches R1B^d^_ub_ to significantly lower values (“go down”, Fig. S3). The reversal of the total Bmi1 concentration to the previous values, which are less than the threshold level P_2_, does not return R1B^d^_ub_ to a high level state. Such reverse switching (“go up”, Fig. S3) occurs only at a second threshold much lower than the first (P_1_, Figs. 2b and S3). Thus, the R1B^d^_ub_ level can be high or low under exactly the same conditions depending on the previous history. Similarly, switching behavior and hysteresis are observed for other Ring1B forms (Figs. 2b and S3). Supplementary Fig. S10 shows bistability plots, which are calculated for an elementary step model and are similar to the plots presented in Fig. 2b for a model that used a MM kinetic description.

Bistability and hysteresis in the Ring1B/Bmi1 network have important ramifications for histone H2A monoubiquitination. Small changes in graded, analog inputs such as the abundance of Bmi1 can convert into digital “On” or “Off” monoubiquitination of histone H2A (Fig. 2d), which would lead to “Off” or “On” expression of H2A-controlled genes. The bistable gene expression choices may be involved in the cell differentiation initiation or other cell fate decisions that are controlled by the changes in the levels of key induced regulators [48]. Interestingly, in case of relatively narrow bistable domains, stochastic variations in the Bmi1 abundance [49], [50] may result in random switching between “Off” or “On” gene expression patterns. This mechanism may contribute to reported bursts of gene expression [51], [52].

### Ring1B Degradation Rates and Ring1B Catalytic Activity Can Display Oscillations and Excitable, Overshoot Transitions

#### Oscillatory dynamics

**Figure 3:**
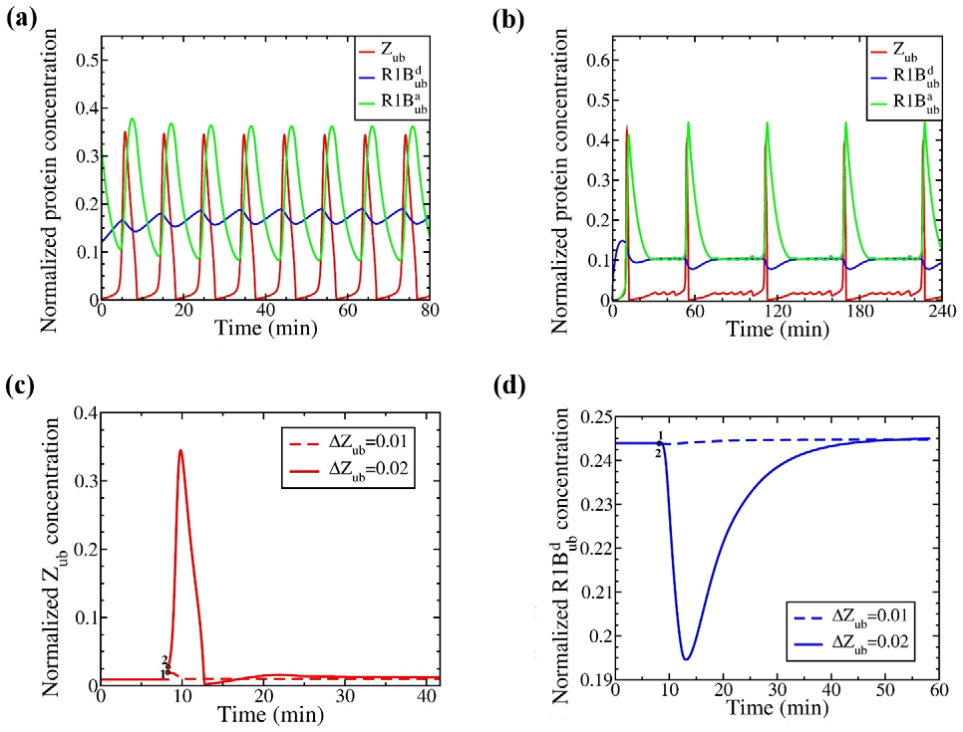
Oscillatory and excitable behavior of the Ring1B/Bmi1 system. (**a**) **and** (**b**) Oscillatory temporal dynamics of Z_ub_ (catalytically active form), R1B^d^_ub_ (targeted for degradation form), and R1B^a^_ub_ (catalytically active form) are brought about by a two-fold and a three-fold increase in the Ring1B deubiquitinase abundance (or catalytic activity): (a) [USP7_tot_] = 2 and [Bmi1_tot_] = 3.25; (b) *k*_1_ = 0.0016, [USP7_tot_] = 3, and [Bmi1_tot_] = 5. (**c**) **and** (**d**) Excitable behavior of the Ring1B/Bmi1 system in response to perturbations to the initial concentrations of Z_ub_ for [USP7_tot_] = 2 and [Bmi1_tot_] = 3. Initially, the system resides in a stable, but excitable steady state (shown by solid line, until the start of perturbations at time t = 500 s). At time t = 500 s, a small perturbation (ΔZ_ub_) of the magnitude 0.01 (point 1) or 0.02 (point 2) is applied to the initial steady-state level of Z_ub_. Temporal responses of (**c**) Z_ub_ and (**d**) R1B^d^_ub_ to a sub-threshold perturbation (point 1) or to an over-threshold perturbation (point 2) are shown by dashed and solid lines, respectively. The remaining parameter values are listed in Table S1. Note that on the timescale of an hour, the oscillatory and excitable behaviors of two systems (where protein synthesis and degradation are neglected or considered) remain the same.

In addition to bistability the interaction circuitry of the Ring1B/Bmi1 system coupled with Ring1B self-ubiquitination and saturability of the deubiquitination reaction can bring about oscillatory and excitable behavior of Ring1B (de)ubiquitination and, thereby, H2A monoubiquitination. Different dynamics of Ring1B degradation and catalytic rates occur in distinct ranges of kinetic parameters. For instance, whereas bistable switches of Ring1 activity occurred for Bmi1 abundances between about 200 and 300 nM and kinetic parameter values given in Table S1, a two-fold increase in DUBs activity (e.g., the USP7 level) and larger Bmi1 abundance lead to self-perpetuating oscillations of Ring1B activity (Fig. 3a). Interestingly, oscillations of R1B^d^_ub_ and R1B^a^_ub_ are out of phase with each other (Fig. 3), because E6-AP-mediated and self-mediated Ring1B ubiquitinations are mutually exclusive [3]. Oscillations in R1B^a^_ub_ slightly lag behind oscillations in the complex Z_ub_, because of the delay caused by R1B^a^_ub_ dissociation from Z_ub_. Overall, periodic declines of the targeted for degradation Ring1B form correspond to increases in Ring1B monoubiquitinating activity and vice versa. Depending on the value of the parameters, the period of these oscillations can vary from minutes to hours (Fig. 3 a,b). Importantly, these features of oscillatory dynamics are also observed for a model at the elementary step level, which does not use the QSS approximation and MM kinetics (Supplementary Fig.S11). These results suggest that these intricate dynamic behaviors are inherent properties of the Ring1B/Bmi1/H2A system and not an artifact of a particular modeling framework used to analyze the system.

#### Excitable response behavior

When the Ring1B/Bmi1 network is not very far from oscillatory regimes, a transient perturbation to a single stable steady state may cause Ring1B activity and degradation rate to overshoot before returning to the basal stationary level. In this case, the Ring1B ligase behaves as an excitable device with a built-in excitability threshold. For sub-threshold perturbations, responses of various Ring1B forms remain small, which is illustrated in Figs. 3c and 3d for a small perturbation of initial steady-state concentrations (dashed lines). However, for over-threshold perturbations, degradable and active Ring1B fractions undergo large excursions and generate high-amplitude overshoots, before returning to the same basal steady state (solid lines). Importantly, there are no intermediate responses merely proportional to the stimulus. Different Ring1B fractions, such as degradable R1B^d^_ub_ and catalytically active Z_ub_ and R1B^a^_ub_, may display different recovery periods returning to the initial steady state. Fig. 3d shows the corresponding rapid descent of R1B^d^_ub_ levels followed by slow recovery after the over-threshold perturbation. Note that excitable behaviors also occur in response to perturbations to systems parameters rather than initial concentrations (Fig. S4).

A quick pulse of active Ring1B concentration enabled by the excitable responses of the Ring1B/Bmi1 network may be particularly useful for the cell to quickly and temporally suppress the expression of genes which are poised for expression by having RNA polymerase bound to the promoter but H2A ubiquitination hindering the polymerase to transcribe the gene. Such situations are found in stem cells and participate in the regulation of differentiation [8]. In fact, our model predicts excitable responses of monoubiquitinated H2A to small changes in active Ring 1B, where over-threshold perturbations induce a large overshoot in H2A monoubiquitination before returning to the stable steady state (supplementary Fig. S4c,d).

Interestingly, this excitable behavior of the Ring1B/Bmi1 network parallels - on a different timescale - the dynamics of classic excitable systems, such as nerve axon [53], heart Purkinje fibres [54], and human atrial cells [55]. In these systems excitable behavior causes the initiation and the propagation of electrical waves. Excitable spikes of Ring1B activity may cause a rapid propagation of H2A ubiquitination across the genome, ensuring the efficient shut-down of transcription.

#### Revealing different dynamics by partitioning the parameter space

**Figure 4:**
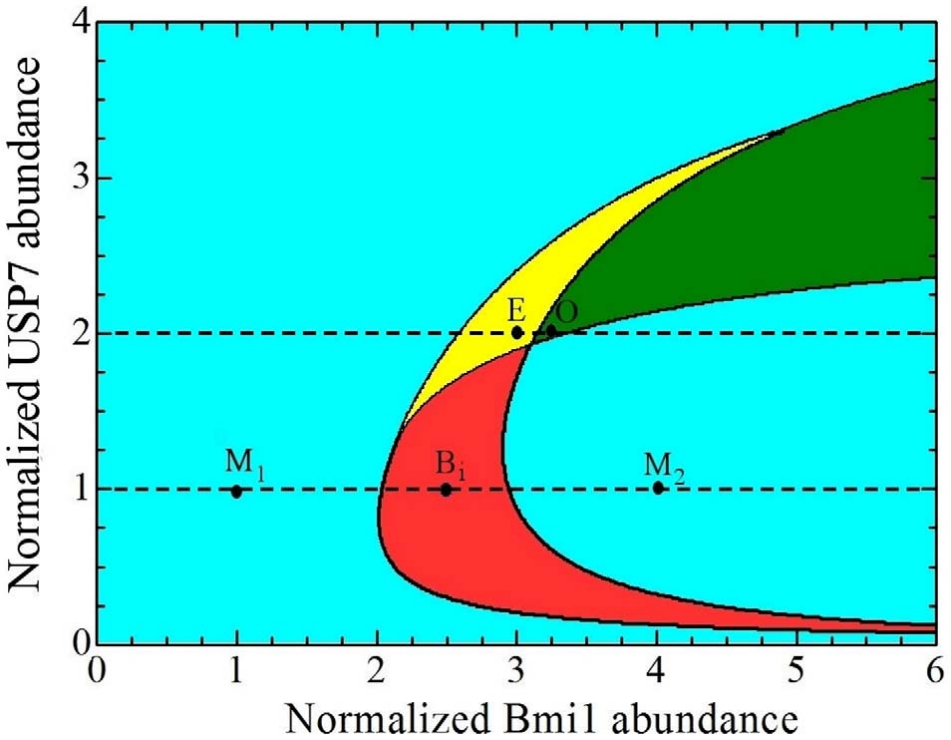
Control of the intricate Ring1B dynamics by Bmi1 and DUB abundances. Stability analysis of the kinetic model reveals the Ring1B/Bmi1 system displays diverse dynamics depending on the abundances of Bmi1 and the Ring1B deubiquitinase USP7: monostable region where a single stable steady state exists (cyan); bistable region where two stable states are separated by an unstable state (red); oscillatory region where a single unstable steady state exists (green); excitable region where a single stable and two unstable steady states exist (yellow). Within a part of the excitable region (close to the oscillatory region), the oscillatory behavior can also be displayed when the initial conditions and ensuing trajectories are out of the basin of attraction of a stable, excitable steady state (see Fig. S5). Points M_1,_ M_2_, B_i_ (i = 1,3), O, and E correspond to the parameter values used in Figs. 2 and 3 and show monostable (M), bistable (B_i_), oscillatory (O), and excitable (E) dynamic behaviors (the different dynamics were classified based on the number and type of eigenvalues and validated by numerical integration, see SI for further details, parameters are given in Table S1).

The dependence of the Ring1B/Bmi1 network dynamics on kinetic parameters can be conveniently described by dividing a plane of two selected parameters into areas, which represent different types of dynamic responses. This partitioning of the parameter space helps us perceive how changes in the Bmi1 and DUB abundances (activity) affect the network dynamics and bring about oscillations, pulses and toggle switches in Ring1B degradation and activity rates (Fig. 4). At very low Bmi1 abundance, monostability is the dominant dynamics. Upregulation of Bmi1 gives rise to more complex behaviors including bistable, oscillatory and excitable responses. For example, at USP7 abundance of 100 nM (Fig. 4), a gradual increase in Bmi1 abundance moves the network from a single stable state (point M_1_) to a bistable regime (point B) and then to another monostable regime (point M_2_). Fig. 4 illustrates that bistability occurs over a large range of USP7 activity (*>*100 nM), and at each USP7 level the Bmi1 abundance can vary over a wide range (>100 nM), suggesting that a bistable behavior is a robust feature of the Ring1B/Bmi1 network. At 200 nM USP7, a gradual increase in the Bmi1 abundance transfers the network from a non-excitable to an excitable, stable state (point E) and then to an oscillatory regime (point O). Thus, under the proper conditions, when both Bmi1 and USP7 are upregulated, self-perpertuating oscillatory responses of Ring1B monoubiquitination activity are facilitated.

## Discussion

Ring1B-dependent monoubiquitination of histone H2A, enhanced by Bmi1, is an essential mechanism of Polycomb-mediated gene silencing. Monoubiquitinated histone H2A is involved in the initiation and maintenance of the silenced state of PRC1 target genes. To understand the temporal dynamics of H2A-directed gene silencing, it is crucial to understand dynamics of the Ring1B/Bmi1 system and H2A monoubiquitination. The present paper shows that complex dynamic behaviors can be brought about by the intrinsic circuitry of Ring1B-Bmi1 interactions, auto-ubiquitination of Ring1B and its ubiquitination and deubiquitination by other ubiquitin ligases and DUBs. Using computational modelling to elucidate these dynamic properties, we demonstrate that the Ring1B/Bmi1 system can act as analog-digital converter, generating abrupt switches, multistable dynamics, oscillations and overshoots. Distinct types of responses facilitate signal discrimination and allow the Ring1B/Bmi1 system to differentially affect gene silencing, which may trigger different cell fates.

We show that overexpression or mutation of Bmi, other ubiquitin ligases and DUBs do not merely change the amplitude of responses to external stimuli, but can dramatically transform the response dynamics. For instance, upregulation of Bmi1 abundance can move the system from a monostable regime prevailing at low Bmi1 levels into more complex regimes exhibiting self-perpertuating oscillatory or bistable responses. Under conditions where the concentrations of Bmi1 and DUBs deubiquitinating Ring1B (e.g. USP7) are high, oscillatory responses are promoted; whereas underexpression of USP7 confers the system more prone to bistable responses.

We show that these complex dynamics of the Ring1B system arise from positive feedback loops brought about by intermolecular self-induced ubiquitination of Ring1B combined with the saturable kinetics of the Ring1B deubiquitinating reactions. Interestingly, additional mechanisms exist in the Ring1B/Bmi1 system, which can give rise to bistable and oscillatory responses. For example, three or more ubiquitination/deubiquitination cycles occurring during the formation of polymeric ubiquitin chains, can bring about bistable behavior provided different ubiquitination forms compete for the same ligase or DUB [21]. Although we confined our analysis to bistable behavior, relaxing the assumption about the first order DUB kinetics brings about multistable behavior. In fact, when both steps 7 and 10 are saturable, multistable steady states can occur in the Ring1B/Bmi1 system. In this case, up to three stable states can be observed for a given total Bmi1 concentration (Fig. S6). However, if positive feedback loops are absent, only abrupt switches, but not bistability can be observed (Fig. S7).

When protein synthesis and degradation rates are explicitly taken into account by our long-timescale model, the intricate dynamic features of the Ring1B/Bmi1 system discussed above, including bistable and oscillatory behaviors, remain the same on short timescales (Fig. S8). As can be seen in Fig. S8, on the timescale up to one hour, a model that describes the long-term dynamics behaves practically indistinguishable from a short-timescale model that neglects protein synthesis and degradation, and both bistable (Figs. S8a) and oscillatory (Figs. S8b) responses are observed. However, on the long timescale (>>1 hr), complex dynamics such as bistability and oscillations might not be exhibited due to the effect of protein synthesis and degradation, and the system would approach a stable steady state after more than 10 hrs (at selected parameter values). The timescale of the experiments exploring the post-translational, (de)ubiquitination dynamics of the Ring1b/Bmi1 system was much less than an hour, which is significantly shorter that the timescale associated with Ring1B/Bmi1 synthesis and degradation (about 3 hour half-life for Ring1B and Bmi1 and ∼7–8 hour half-life for the Ring1B-Bmi1 complex) [18]. Accordingly, we have mainly focused on the one hour timescale to account for the data on different ubiquitinated forms of Ring1B and Bmi1 that relaxed to (quasi-) stationary concentrations [18]. Further experimental testing of the model requires the kinetic monitoring of different forms of ubiquitin chains that control Ring1B activity or target it for degradation. However, at present, there are no reagents that can discern between these different linkage types. We were able to obtain experimental data that support some modelling predictions, but are not fully conclusive to claim that the predicted dynamics (switches, oscillations or excitable response) are realized in the tested cells (Supplementary Text S1, section 6, and Figs. S12, S13, S14). Although we are still carrying out further experiments, the more complete verification of model predictions requires extensive time and effort that goes beyond the scope of the current paper. Because this is the first mathematical model of the Ring1B/Bmi1 ubiquitination system, our main objective is to draw attention to a rich repertoire of dynamical behaviors that the ubiquitination system can exhibit.

The results of the present study shed light on recent experimental findings related to concentrations of Bmi1 and ubiquitinated histone H2A in stem cells, tumour cells and cells undergoing differentiation. Among the PRC1 component proteins, Bmi1 has been demonstrated to be strongly involved in multiple biological processes including tumorigenesis, stem cells self-renewal, and differentiation [6]. Overexpression of Bmi1 is frequently observed in various types of human cancers, including lung cancer, ovarian cancer, acute myeloid leukemia, nasopharyngeal carcinoma, and neuroblastoma [56], [57], [58], [59], [60]. This oncogenic property of Bmi1 has been linked to its ability to protect cells from apoptosis through suppressing the expression of tumour suppressor and pro-apoptotic genes. For examples, in Bmi1-deficient mice the number of lymphocytes is significantly reduced due to increased apoptosis [61]. Expression of Bmi1 in stem cells leads to the silencing of the tumour suppressor locus *CDKN2A*, which encodes INK4A and ARF [6]. These observations are consistent with our model predictions that overexpression of Bmi1 upregulates H2A monoubiquitination, which facilitates gene silencing.

Bmi1 is highly expressed in adult and fetal mouse and adult human hematopoietic stem cells (HSCs) [62], suggesting its important roles in maintaining the stem cell pool. Indeed, hematopoietic capacity is markedly reduced in Bmi1-knock out mice because of defective self-renewal ability of HSCs [62]. Downregulation of Bmi1 also results in decreased proliferation and self-renewal ability both *in vitro* and *in vivo* of neural stem cells [63]. Interestingly, Hosen et al. [64] showed that Bmi1 expression is high in HSCs and that Bmi1 is downregulated once the HSCs have differentiated into a particular lineage. Such differentiation in HSC cells may be enabled by the irreversible toggle switch characteristics of the Ring1B/Bmi1 system revealed in this paper.

It has been previously shown that shortly after murine erythroleukemia (MEL) cells were exposed to inducers of differentiation, significant increase of histone H2A ubiquitination occurred before returning to control levels [65]. Such transient pulse of monoubiquitinated H2A, which appears essential for MEL differentiation, can be explained by H2A excitability that follows gradual activation by some upstream regulators (e.g. Ring1B, Bmi1, external ligases or DUBs). The elevation of histone H2A monoubiqutination may initiate silencing of inhibitors of differentiation genes and thereby instigate differentiation. This implies that the Ring1B/Bmi1 excitable behavior can be implicated in cell-fate decision processes.

Our model can also helps explaining *in vivo* data involving knockout of E6-AP, the main exogenous ligase modifying Ring1B for degradation. E6-AP knockout mice display an elevated level of Ring1B and ubiquitinated histone H2A in various tissues, including cerebellar Purkinje neurons and liver.

When the number of Ring1B/Bmi1 molecules is not very high, random variation (noise) in these protein numbers influence signaling dynamics [48]. Within the bistable regime, depending on the stimulus history, intrinsic or extrinsic noise can lead to abrupt, random switches between low and high states of ubiquitinated H2A, potentially resulting in switching between On and Off states of H2A-silencing genes, respectively. This might partly contribute to transcriptional bursts of expression of these genes [66].

In summary, this paper presents a computational model of the Ring1B/Bmi1 ubiquitination system that reveals a high intrinsic complexity of the dynamics of Ring1B activity and H2A monoubiquitination and allows for direct experimental testing. Our findings provide a new perspective on (de)ubiquitination networks, which can display remarkably rich and complex dynamic behaviors.

## Supporting Information

Figure S1 Temporal dynamics of R1B^d^_ub_ corresponding to the monostable regions (cf. Figure.2b in the main text). Temporal dynamics of [R1B^d^_ub_] approaching steady states M_1_ and M_2_ in the monostable regions ([Bmi1_tot_] = 1 and 4 respectively) for two different initial conditions: (solid line) [Bmi1] = 1, [R1B] = 1, and the remaining initial concentrations equal to zero; (dashed line) [Bmi1] = 4, [R1B] = 1, and the remaining concentrations equal to zero.
(TIF)

Figure S2 Bistability and hysteresis in the Ring1B/Bmi1 system. Dependence of the steady state levels of R1B^a^_ub_ (red) and R1B_ub_ (blue) on the Bmi1 abundance. Stable and unstable steady states are shown by solid and dotted lines, respectively. Turning points P_1_ and P_2_ indicate saddle-node bifurcations.
(TIF)

Figure S3 Hysteresis and biological memory. Dependence of the steady state R1B^d^_ub_ levels on the Bmi1 abundance. Stable and unstable steady states are shown by solid and dotted lines respectively. The system resigning in the high or low R1B^d^_ub_ states retains the corresponding state “memory” until the threshold in Bmi1 abundance is reached. These thresholds correspond to the turning points P_1_ and P_2_, which are saddle-node bifurcations and related to “go up/down” switches.
(TIF)

Figure S4 Excitable behavior of the Ring1B/Bmi1 system in response to perturbations. (**a**) and (**b**). Initially, the system resides in a stable, but excitable steady state (horizontal solid line) until a 40% perturbation to the initial parameter value *k_6a_* starts at time t = 500 s and continue for 80 or 85 seconds. Temporal responses of H2A_ub_ to a sub-threshold perturbation (of 80 sec duration) and to an over-threshold perturbation (of 85 sec duration) are shown by dashed and solid lines, respectively, for (**a**) Z_ub_ and (**b**) R1B^d^_ub_. (**c**) and (**d**) Excitable behavior of monoubiquitinated H2A (H2A_ub_) in response to perturbations to the concentrations of active Ring 1B form (Z_ub,_ panel **c**) and parameters (*k*_6a_, panel **d**). (**c**) Initial stable steady state is shown by horizontal solid line. At time t = 8.3 min a small perturbation (ΔZ_ub_) is applied to Z_ub_. The temporal responses of H2A_ub_ resulting from a sub-threshold or an over-threshold perturbation are shown by dashed and solid lines, respectively. Since the total protein concentration is altered after increasing Z_ub_, the H2A_ub_ steady states are slightly different compared to the unperturbed states. (**d**) A 40% perturbation in the initial *k*_6a_ value was applied for 80 sec or 85 sec. Temporal responses of H2A_ub_ to a sub-threshold perturbation (of 80 sec duration) and to an over-threshold perturbation (of 85 sec duration) are shown by dashed and solid lines, respectively.
(TIF)

Figure S5 Oscillatory behavior displayed in the excitable region. Temporal dynamics of R1B^d^_ub_ that shows sustained oscillatory or monostable behaviors, depending on the initial conditions. For the initial concentrations [USP7] = 2, [Bmi1^d^_ub_] = 3.1, and [R1B] = 1, R1B^d^_ub_ displays a single stable steady state (dashed line), whereas for the initial concentratrations [USP7] = 2, [Bmi1] = 3.1, [R1B] = 1, R1B^d^_ub_ displays self-perpetuating oscillations (solid line). All the remaining initial concentrations equal to zero.
(TIF)

Figure S6 Multistability in the Ring1B/Bmi1 ubiquitination system. Dependence of the steady state levels of Z_ub_ (catalytically active) on the Bmi1 abundance when both reactions 7 and 10 follow the Michaelis-Menten kinetics. Here *v*_10_ = *k*_10_ [R1B_ub_]/(*K*_M10_ +[ R1B_ub_]), *K*_M10_ = 0.1, *k*_6a_ = 5 s^−1^, and *k*_11_ = 0.002 s^−1^. The remaining normalized parameters are given in Table S1. Stable and unstable steady states are shown by solid and dotted lines respectively.
(TIF)

Figure S7 Ultrasensitive behavior when positive feedback loops are absent. Dependence of the steady state levels of Z_ub_ (catalytically active form, red curve) and R1B^d^_ub_ (targeted for degradation form, blue) on the Bmi1 abundance for the same parameters as in Figure. 2b except for *k*_6_ = *k*_9_ = 20 s^−1^, *k*_6a_ = *k*_9a_ = 0, and *v*_10_ = *k*_10_ [R1B_ub_]/(*K*_M10_ +[ R1B_ub_]) with *K*_M10_ = 0.01.
(TIF)

Figure S8 Comparison of the [R1B^d^_ub_] temporal dynamics on the short- and long-timescales. (**a**) Bistable behavior at short-timescale. Protein synthesis and degradation are included (magenta) or neglected (blue) at short- and long-timescales for two different initial conditions: [Bmi1^d^_ub_] = 2.5, [R1B] = 1 (solid lines); [Bmi1] = 2.5, [R1B] = 1 (dashed lines); the remaining initial concentrations equal to zero. (**b**) Oscillatory behavior at short times. Protein synthesis and degradation are included (magenta) or neglected (blue) at short and long timescales for the initial condition: [Bmi1^d^_ub_] = 3.25, [R1B] = 1 and the remaining concentrations equal to zero. On short timescales (0–60 minutes) the system that includes protein synthesis and degradation behaves almost identically to the system where synthesis and degradation are neglected. In contrast, on long timescales (>>1 hour) when synthesis and degradation are included, a unique steady state (whose value depends on the synthesis and degradation rates) is reached.
(TIF)

Figure S9 Reactions scheme of a mass-action description of the deubiquitinase USP7. Here, deubiquitination of Z_ub_ into Z, catalysed by the deubiquitinase USP7, is explicitly modelled using elementary reactions (7 and 7′) as opposed to the lumped reaction with MM kinetics (reaction 7 in Figure. 1, main text). This new mass-action model is described by equations given below and in Table S2.
(TIF)

Figure S10 Bistability and hysteresis in the Ring1B/Bmi1 system revealed by the mass-action model when the MM kinetics is inapplicable. (**a**) Dependence of the steady-state levels of Z_ub_ and R1B^a^_ub_ (catalytically active forms of Ring1B in complex and in free form) on the Bmi1 abundance. Unstable states are shown by dotted lines. (**b**) Dependence of the stationary level of monoubiquitinated H2A_ub_ on the Bmi1 abundance. Parameter values are [USP7_tot_] = 52 nM, [R1B_tot_] = 400 nM, *k*_7f_ = 0.5 nM^−1^ s^−1^, *k*_7r_ = 5 s^−1^, *k*_7cat_ = 1 s^−1^, *k*_4_ = 0.005 nM^−1^ s^−1^, *k*_10_ = 0.0375 nM^−1^ s^−1^, *k*_11_ = 0.025 nM^−1^ s^−1^ and *k*_13_ = 1s^−1^, the remaining parameter values are given in Table S2.
(TIF)

Figure S11 Oscillatory and excitable behavior of the Ring1B/Bmi1 system in the mass-action model. (**a**) Oscillatory temporal dynamics of Z_ub_ (catalytically active form), R1B^d^_ub_ (targeted for degradation form), and R1B^a^_ub_ (catalytically active, free form) when the system is in the MM limit. Parameter values, [USP7_tot_] = 1 nM, [R1B_tot_] = 100 nM, *k*_7f_ = 4 nM^−1^ s^−1^, *k*_7r_ = 0.01 s^−1^, *k*_7cat_ = 1 s^−1^, *k*_4_ = 0.02 nM^−1^ s^−1^, *k*_10_ = 0.15 nM^−1^ s^−1^, *k*_11_ = 0.1 nM^−1^ s^−1^, the remaining parameters are given in Table S2. (**b**) Oscillatory temporal dynamics of Z_ub_, R1B^d^_ub_, R1B^a^_ub_ when the MM kinetics is inapplicable. Parameter values, [USP7_tot_] = 100 nM, [R1B_tot_] = 200 nM, *k*_7f_ = 0.15 nM^−1^ s^−1^, *k*_7r_ = 0.045 s^−1^, *k*_7cat_ = 0.021 s^−1^, *k*_4_ = 0.002 nM^−1^ s^−1^, *k*_10_ = 0.015 nM^−1^ s^−1^, *k*_11_ = 0.005 nM^−1^ s^−1^, the remaining parameter values are given in Table S2. (**c**) Oscillatory temporal dynamics of Z_ub_, R1B^d^_ub_, R1B^a^_ub_ for the same parameter values as in panel **b**, except *k*_7f_ = 0.175 nM^−1^ s^−1^. (**d**) Excitable behavior of the Ring1B/Bmi1 system in response to perturbations. Initially, the system resides in a stable, but excitable steady state (horizontal solid line) until a 40% perturbation to the initial parameter value *k_6a_* starts at time t = 500 s and continues for 5 or 15 seconds. Temporal responses of Z_ub_ to a sub-threshold perturbation (of 5 sec duration) and to an over-threshold perturbation (of 15 sec duration) are shown by dashed and solid lines, respectively. Parameter values, [USP7_tot_] = 100 nM, [R1B_tot_] = 200 nM, [Bmi1_tot_] = 300 nM, *k*_7f_ = 0.15 nM^−1^ s^−1^, *k*_7r_ = 0.045 s^−1^, *k*_7cat_ = 0.021 s^−1^, *k*_4_ = 0.002 nM^−1^ s^−1^, *k*_10_ = 0.015 nM^−1^ s^−1^, *k*_11_ = 0.005 nM^−1^ s^−1^, the remaining parameters are given in Table S2.
(TIF)

Figure S12 Quantified amount of ubiquitinated Ring1B in response to increasing levels of transfected Flag-Ring1B (in µg) at 1 µg of transfected HA-Ubiquitin (raw data are in Figure.S14).
(TIF)

Figure S13 Dependence of steady-state levels of total active ubiquitinated Ring1B (red) and total ubiquitinated Ring1B on increasing concentrations of Ring1B abundance.
(TIF)

Figure S14 Cellular assay for Ring 1B ubiquitination. *Cos-1* cells were co-transfected with the indicated amounts of a FLAG-tagged Ring1B (Flag-R1B) construct and an HA-tagged ubiquitin construct. A. Total lysates were analyzed by Western blotting using antibodies against Flag and HA epitopes. B. Flag immunoprecipitations were performed after normalization of Flag-tagged Ring1B level and were analyzed by Western blotting using antibodies against the HA and Flag epitopes.
(TIF)

Table S1 Rate expressions and kinetic parameters of the Michaelis-Menten model.
(DOC)

Table S2 Rate expressions and kinetic parameters of the Mass-Action model.
(DOC)

Text S1 Derivation of the Michaelis-Menten model and Mass-Action model describing the dynamics of the Ring1B/Bmi1 ubiquitination system and comparison of model predictions (sections 1–3 and 5). Theoretical background on Linear Stability Analysis (section 4) and Preliminary Experimental Validation of Model Predictions (section 6).
(DOC)
