# Supplementary figures and images for: Switches, Excitable Responses and Oscillations in the Ring1B/Bmi1 Ubiquitination System
Source: PLoS Comput Biol. 2011 Dec 15;7(12):e1002317. doi: 10.1371/journal.pcbi.1002317 (PMC3240587; doi:10.1371/journal.pcbi.1002317)

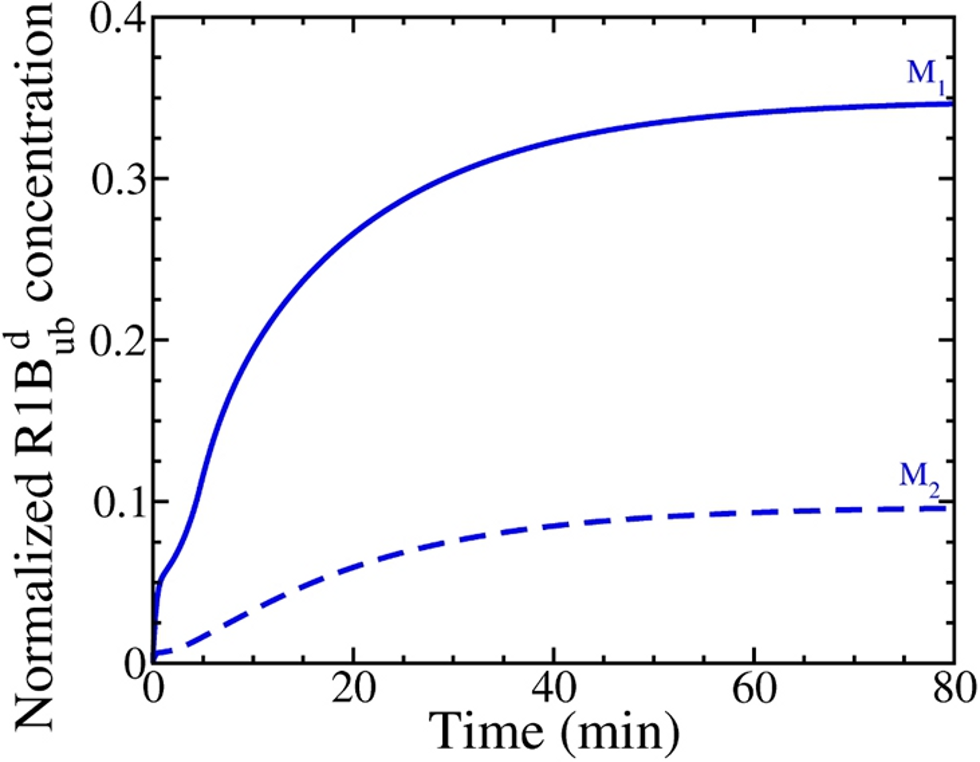

Supplement: Figure S1 — Temporal dynamics of R1Bd ub corresponding to the monostable regions (cf. Figure.2b in the main text). Temporal dynamics of [R1Bd ub] approaching steady states M1 and M2 in the monostable regions ([Bmi1tot] = 1 and 4 respectively) for two different initial conditions: (solid line) [Bmi1] = 1, [R1B] = 1, and the remaining initial concentrations equal to zero; (dashed line) [Bmi1] = 4, [R1B] = 1, and the remaining concentrations equal to zero. (TIF) [file pcbi.1002317.s001.tif]

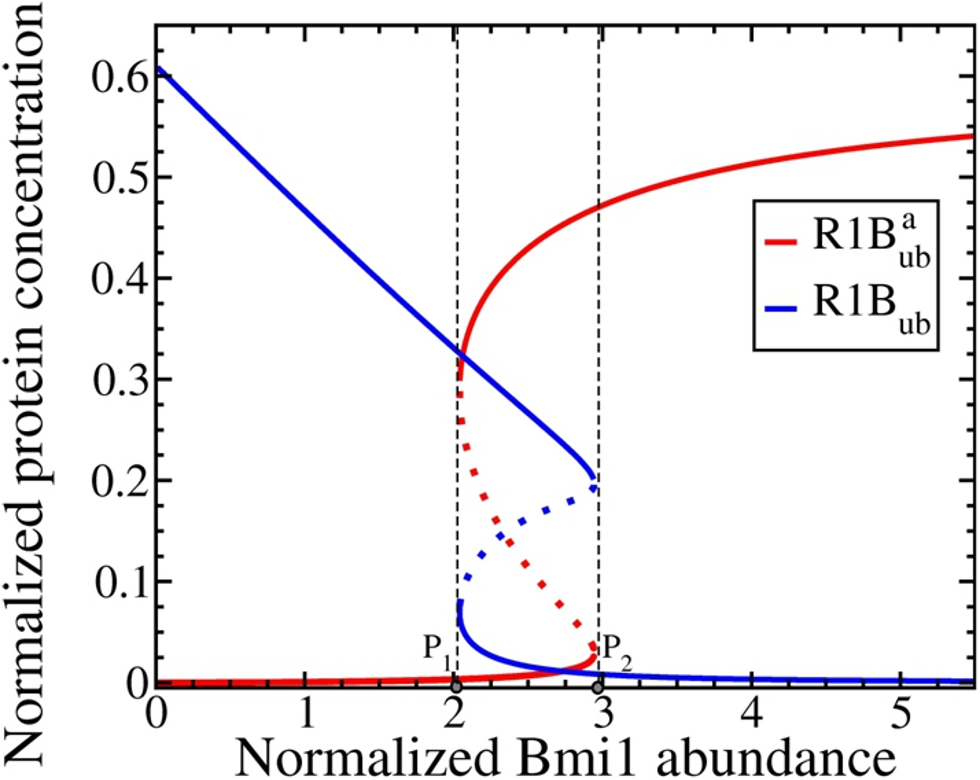

Supplement: Figure S2 — Bistability and hysteresis in the Ring1B/Bmi1 system. Dependence of the steady state levels of R1Ba ub (red) and R1Bub (blue) on the Bmi1 abundance. Stable and unstable steady states are shown by solid and dotted lines, respectively. Turning points P1 and P2 indicate saddle-node bifurcations. (TIF) [file pcbi.1002317.s002.tif]

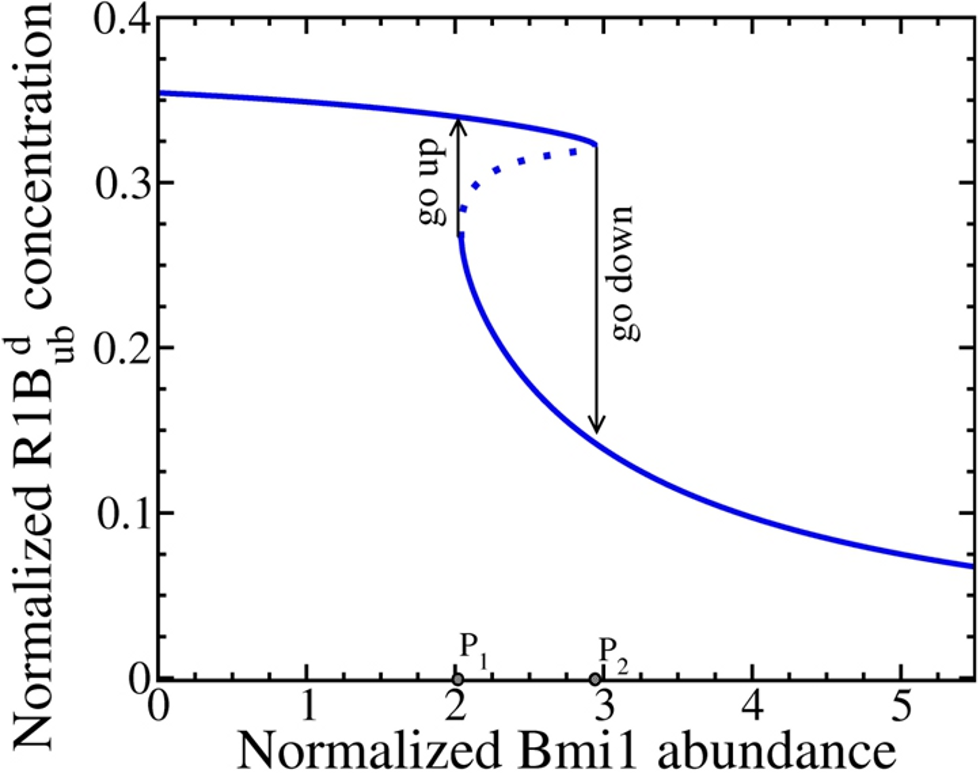

Supplement: Figure S3 — Hysteresis and biological memory. Dependence of the steady state R1Bd ub levels on the Bmi1 abundance. Stable and unstable steady states are shown by solid and dotted lines respectively. The system resigning in the high or low R1Bd ub states retains the corresponding state “memory” until the threshold in Bmi1 abundance is reached. These thresholds correspond to the turning points P1 and P2, which are saddle-node bifurcations and related to “go up/down” switches. (TIF) [file pcbi.1002317.s003.tif]

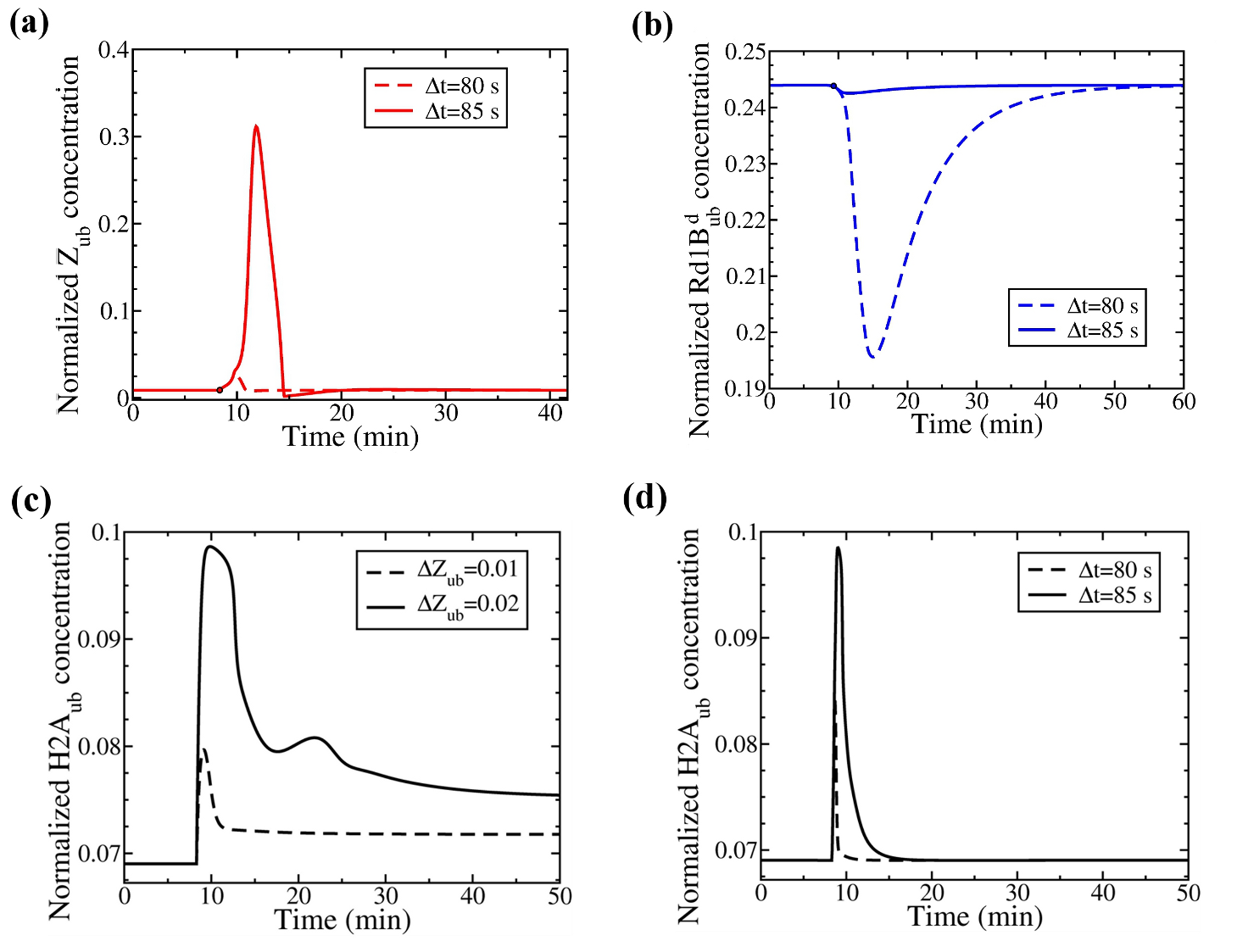

Supplement: Figure S4 — Excitable behavior of the Ring1B/Bmi1 system in response to perturbations. (a) and (b). Initially, the system resides in a stable, but excitable steady state (horizontal solid line) until a 40% perturbation to the initial parameter value k6a starts at time t = 500 s and continue for 80 or 85 seconds. Temporal responses of H2Aub to a sub-threshold perturbation (of 80 sec duration) and to an over-threshold perturbation (of 85 sec duration) are shown by dashed and solid lines, respectively, for (a) Zub and (b) R1Bd ub. (c) and (d) Excitable behavior of monoubiquitinated H2A (H2Aub) in response to perturbations to the concentrations of active Ring 1B form (Zub, panel c) and parameters (k 6a, panel d). (c) Initial stable steady state is shown by horizontal solid line. At time t = 8.3 min a small perturbation (ΔZub) is applied to Zub. The temporal responses of H2Aub resulting from a sub-threshold or an over-threshold perturbation are shown by dashed and solid lines, respectively. Since the total protein concentration is altered after increasing Zub, the H2Aub steady states are slightly different compared to the unperturbed states. (d) A 40% perturbation in the initial k 6a value was applied for 80 sec or 85 sec. Temporal responses of H2Aub to a sub-threshold perturbation (of 80 sec duration) and to an over-threshold perturbation (of 85 sec duration) are shown by dashed and solid lines, respectively. (TIF) [file pcbi.1002317.s004.tif]

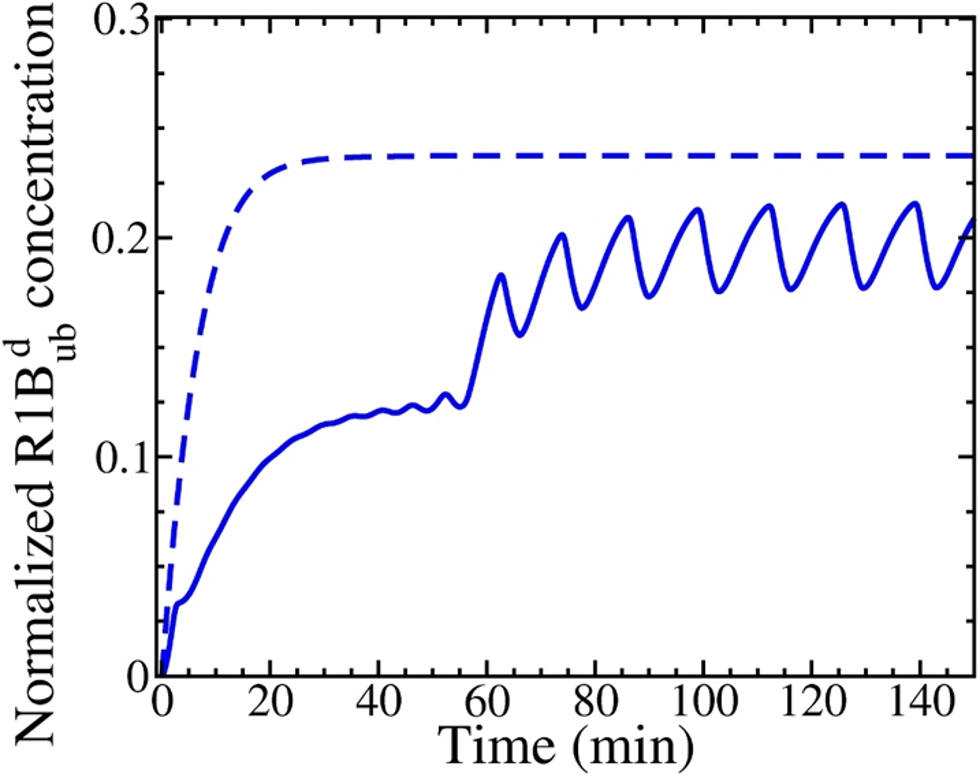

Supplement: Figure S5 — Oscillatory behavior displayed in the excitable region. Temporal dynamics of R1Bd ub that shows sustained oscillatory or monostable behaviors, depending on the initial conditions. For the initial concentrations [USP7] = 2, [Bmi1d ub] = 3.1, and [R1B] = 1, R1Bd ub displays a single stable steady state (dashed line), whereas for the initial concentratrations [USP7] = 2, [Bmi1] = 3.1, [R1B] = 1, R1Bd ub displays self-perpetuating oscillations (solid line). All the remaining initial concentrations equal to zero. (TIF) [file pcbi.1002317.s005.tif]

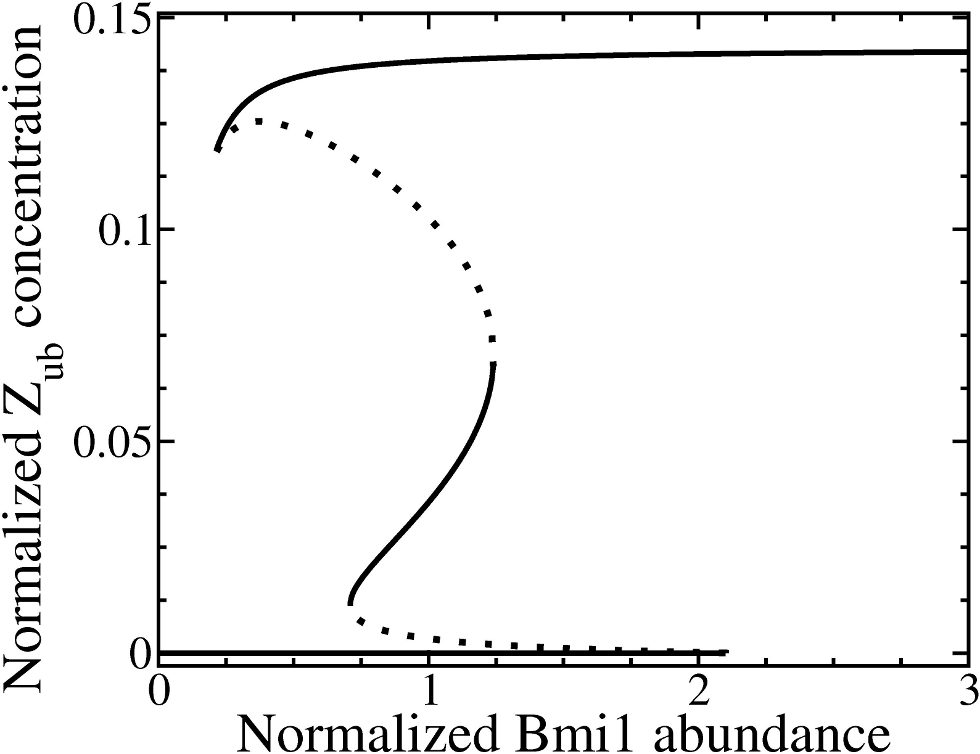

Supplement: Figure S6 — Multistability in the Ring1B/Bmi1 ubiquitination system. Dependence of the steady state levels of Zub (catalytically active) on the Bmi1 abundance when both reactions 7 and 10 follow the Michaelis-Menten kinetics. Here v 10 = k 10 [R1Bub]/(K M10 +[ R1Bub]), K M10 = 0.1, k 6a = 5 s−1, and k 11 = 0.002 s−1. The remaining normalized parameters are given in Table S1. Stable and unstable steady states are shown by solid and dotted lines respectively. (TIF) [file pcbi.1002317.s006.tif]

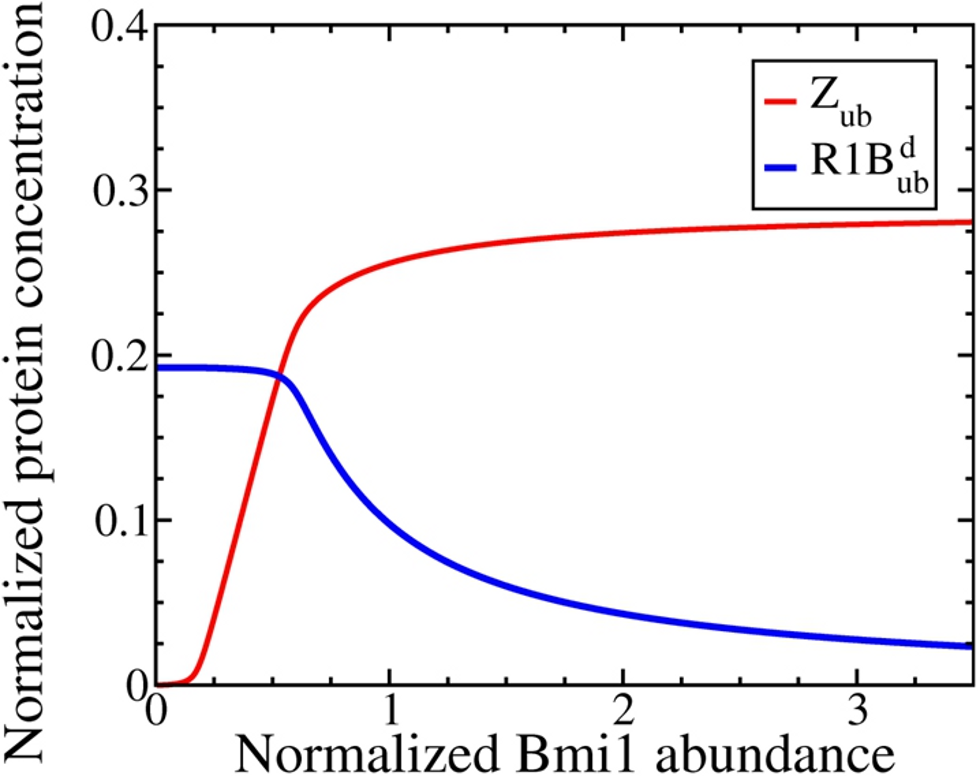

Supplement: Figure S7 — Ultrasensitive behavior when positive feedback loops are absent. Dependence of the steady state levels of Zub (catalytically active form, red curve) and R1Bd ub (targeted for degradation form, blue) on the Bmi1 abundance for the same parameters as in Figure. 2b except for k 6 = k 9 = 20 s−1, k 6a = k 9a = 0, and v 10 = k 10 [R1Bub]/(K M10 +[ R1Bub]) with K M10 = 0.01. (TIF) [file pcbi.1002317.s007.tif]

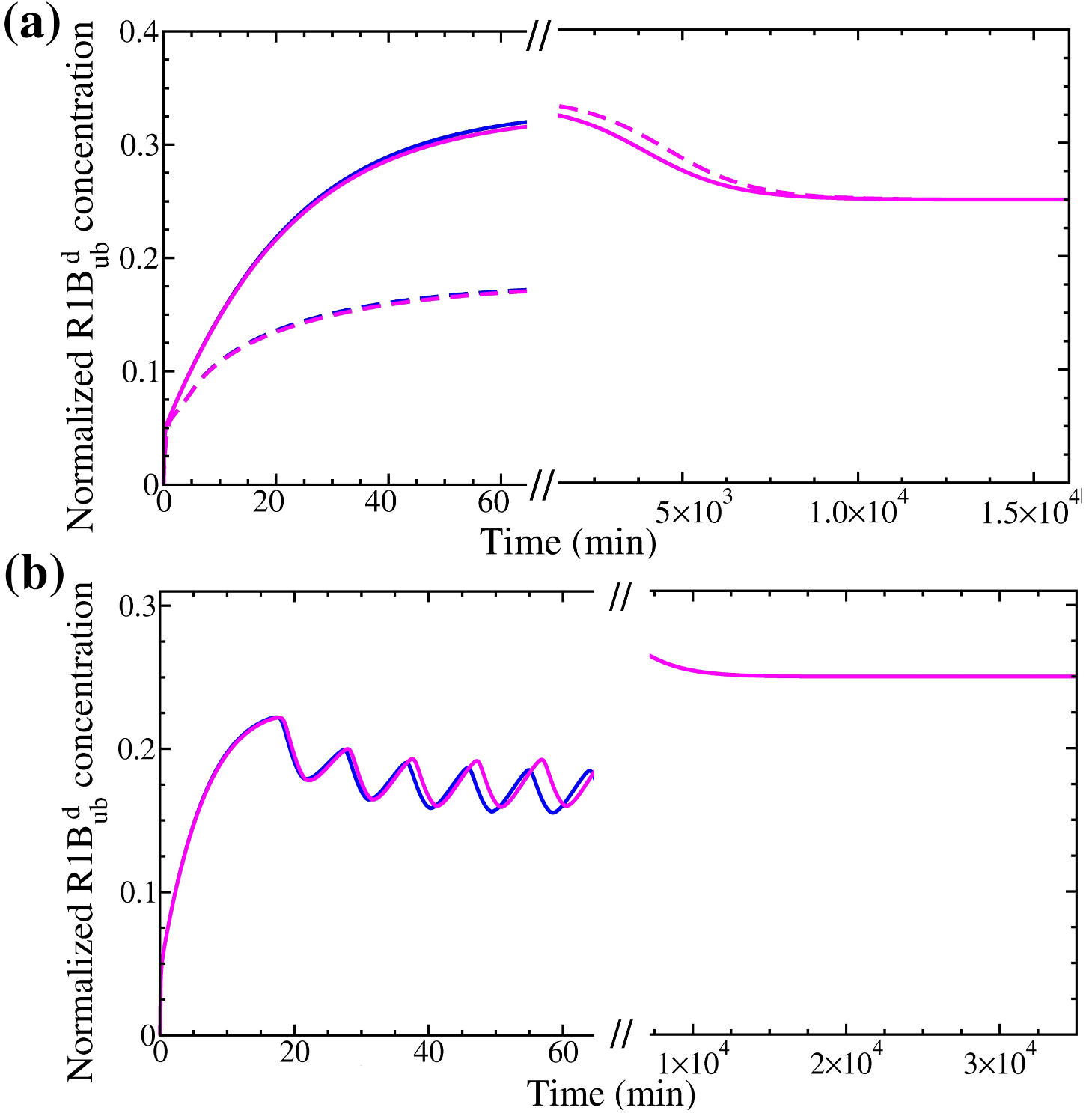

Supplement: Figure S8 — Comparison of the [R1Bd ub] temporal dynamics on the short- and long-timescales. (a) Bistable behavior at short-timescale. Protein synthesis and degradation are included (magenta) or neglected (blue) at short- and long-timescales for two different initial conditions: [Bmi1d ub] = 2.5, [R1B] = 1 (solid lines); [Bmi1] = 2.5, [R1B] = 1 (dashed lines); the remaining initial concentrations equal to zero. (b) Oscillatory behavior at short times. Protein synthesis and degradation are included (magenta) or neglected (blue) at short and long timescales for the initial condition: [Bmi1d ub] = 3.25, [R1B] = 1 and the remaining concentrations equal to zero. On short timescales (0–60 minutes) the system that includes protein synthesis and degradation behaves almost identically to the system where synthesis and degradation are neglected. In contrast, on long timescales (>>1 hour) when synthesis and degradation are included, a unique steady state (whose value depends on the synthesis and degradation rates) is reached. (TIF) [file pcbi.1002317.s008.tif]

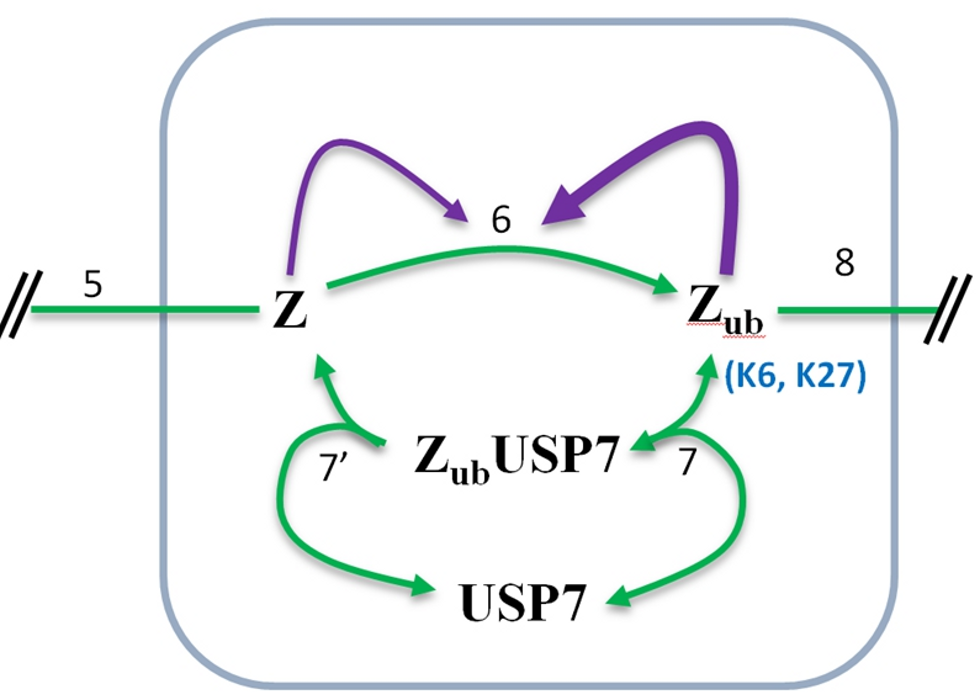

Supplement: Figure S9 — Reactions scheme of a mass-action description of the deubiquitinase USP7. Here, deubiquitination of Zub into Z, catalysed by the deubiquitinase USP7, is explicitly modelled using elementary reactions (7 and 7′) as opposed to the lumped reaction with MM kinetics (reaction 7 in Figure. 1, main text). This new mass-action model is described by equations given below and in Table S2. (TIF) [file pcbi.1002317.s009.tif]

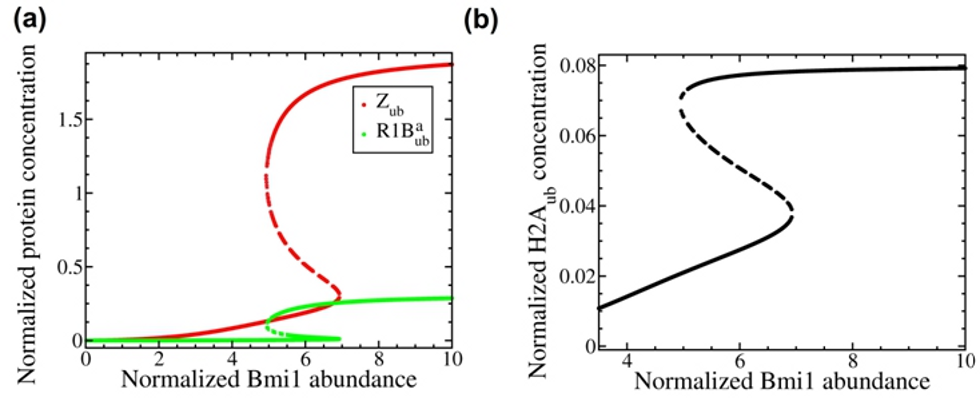

Supplement: Figure S10 — Bistability and hysteresis in the Ring1B/Bmi1 system revealed by the mass-action model when the MM kinetics is inapplicable. (a) Dependence of the steady-state levels of Zub and R1Ba ub (catalytically active forms of Ring1B in complex and in free form) on the Bmi1 abundance. Unstable states are shown by dotted lines. (b) Dependence of the stationary level of monoubiquitinated H2Aub on the Bmi1 abundance. Parameter values are [USP7tot] = 52 nM, [R1Btot] = 400 nM, k 7f = 0.5 nM−1 s−1, k 7r = 5 s−1, k 7cat = 1 s−1, k 4 = 0.005 nM−1 s−1, k 10 = 0.0375 nM−1 s−1, k 11 = 0.025 nM−1 s−1 and k 13 = 1s−1, the remaining parameter values are given in Table S2. (TIF) [file pcbi.1002317.s010.tif]

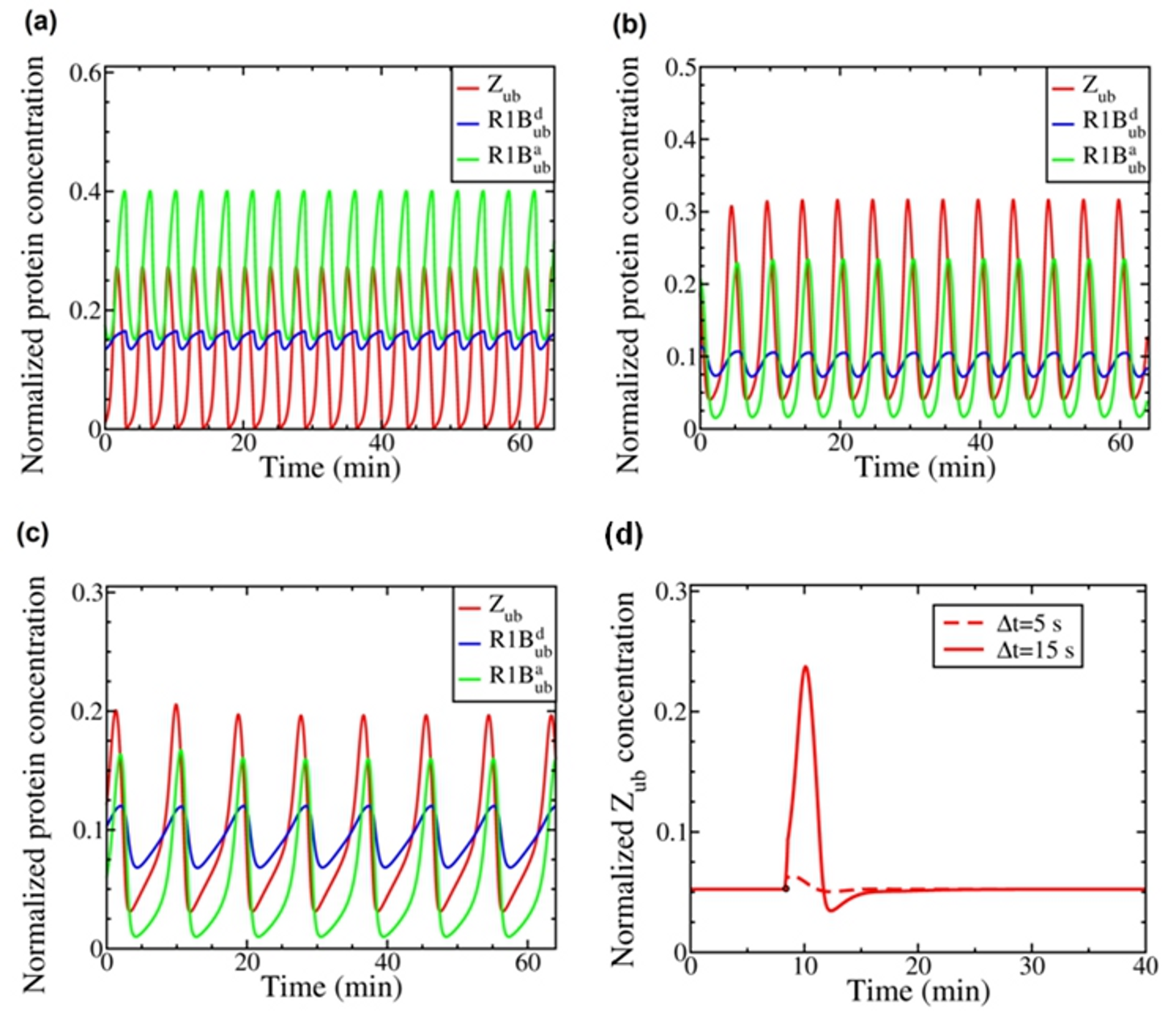

Supplement: Figure S11 — Oscillatory and excitable behavior of the Ring1B/Bmi1 system in the mass-action model. (a) Oscillatory temporal dynamics of Zub (catalytically active form), R1Bd ub (targeted for degradation form), and R1Ba ub (catalytically active, free form) when the system is in the MM limit. Parameter values, [USP7tot] = 1 nM, [R1Btot] = 100 nM, k 7f = 4 nM−1 s−1, k 7r = 0.01 s−1, k 7cat = 1 s−1, k 4 = 0.02 nM−1 s−1, k 10 = 0.15 nM−1 s−1, k 11 = 0.1 nM−1 s−1, the remaining parameters are given in Table S2. (b) Oscillatory temporal dynamics of Zub, R1Bd ub, R1Ba ub when the MM kinetics is inapplicable. Parameter values, [USP7tot] = 100 nM, [R1Btot] = 200 nM, k 7f = 0.15 nM−1 s−1, k 7r = 0.045 s−1, k 7cat = 0.021 s−1, k 4 = 0.002 nM−1 s−1, k 10 = 0.015 nM−1 s−1, k 11 = 0.005 nM−1 s−1, the remaining parameter values are given in Table S2. (c) Oscillatory temporal dynamics of Zub, R1Bd ub, R1Ba ub for the same parameter values as in panel b, except k 7f = 0.175 nM−1 s−1. (d) Excitable behavior of the Ring1B/Bmi1 system in response to perturbations. Initially, the system resides in a stable, but excitable steady state (horizontal solid line) until a 40% perturbation to the initial parameter value k6a starts at time t = 500 s and continues for 5 or 15 seconds. Temporal responses of Zub to a sub-threshold perturbation (of 5 sec duration) and to an over-threshold perturbation (of 15 sec duration) are shown by dashed and solid lines, respectively. Parameter values, [USP7tot] = 100 nM, [R1Btot] = 200 nM, [Bmi1tot] = 300 nM, k 7f = 0.15 nM−1 s−1, k 7r = 0.045 s−1, k 7cat = 0.021 s−1, k 4 = 0.002 nM−1 s−1, k 10 = 0.015 nM−1 s−1, k 11 = 0.005 nM−1 s−1, the remaining parameters are given in Table S2. (TIF) [file pcbi.1002317.s011.tif]

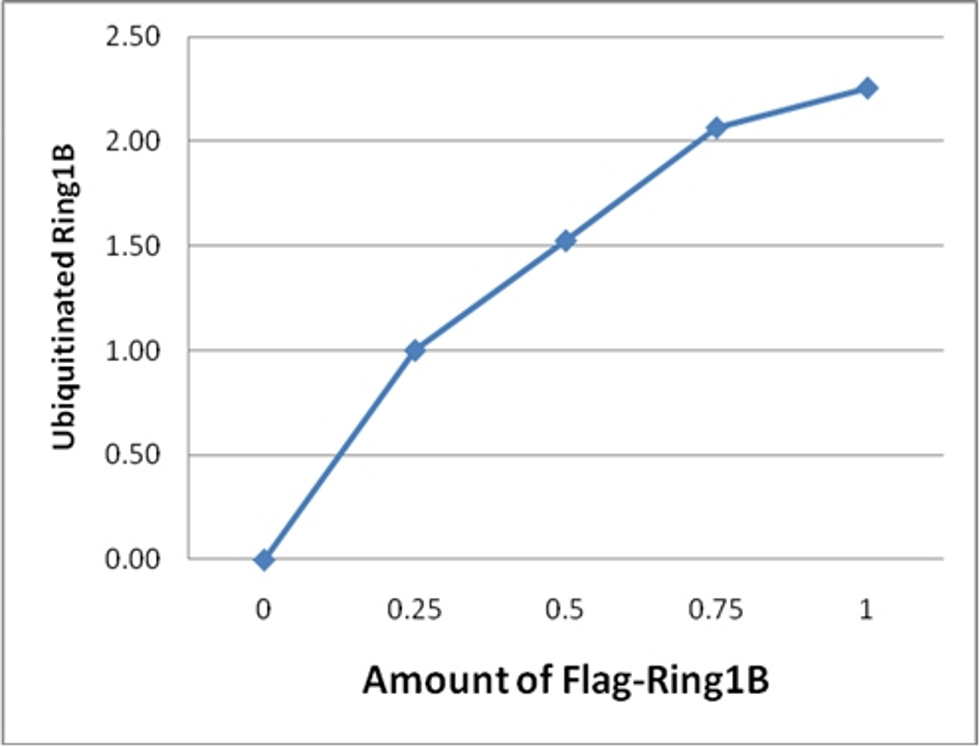

Supplement: Figure S12 — Quantified amount of ubiquitinated Ring1B in response to increasing levels of transfected Flag-Ring1B (in µg) at 1 µg of transfected HA-Ubiquitin (raw data are in Figure.S14). (TIF) [file pcbi.1002317.s012.tif]

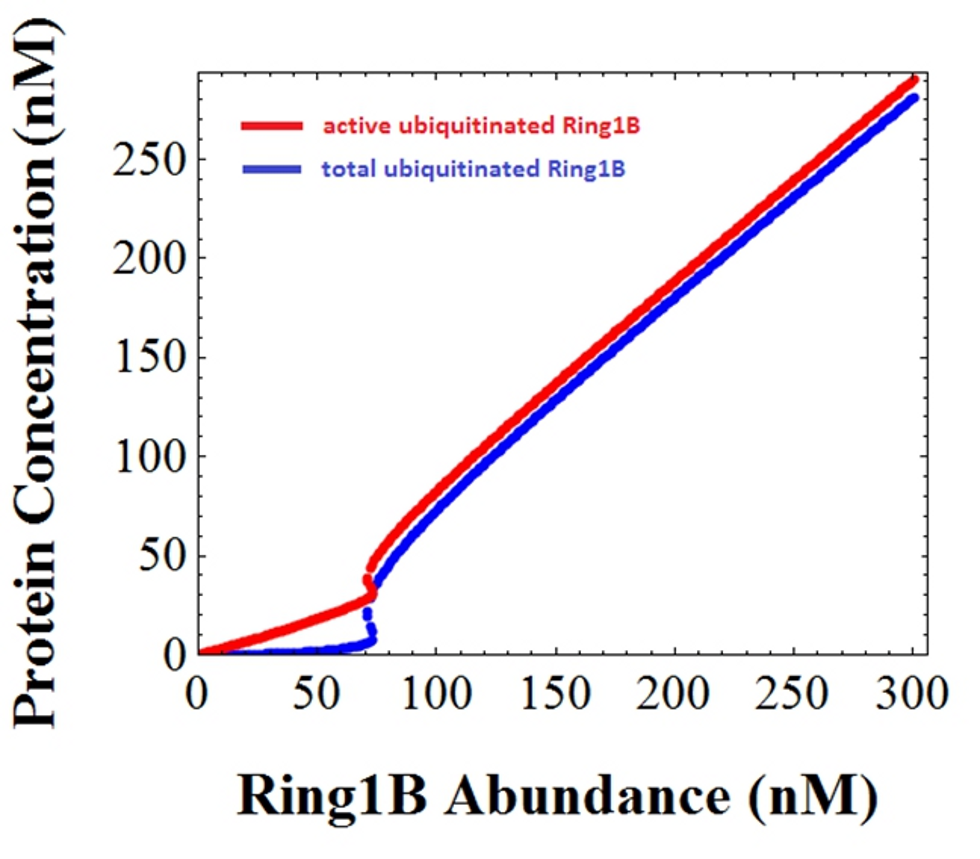

Supplement: Figure S13 — Dependence of steady-state levels of total active ubiquitinated Ring1B (red) and total ubiquitinated Ring1B on increasing concentrations of Ring1B abundance. (TIF) [file pcbi.1002317.s013.tif]

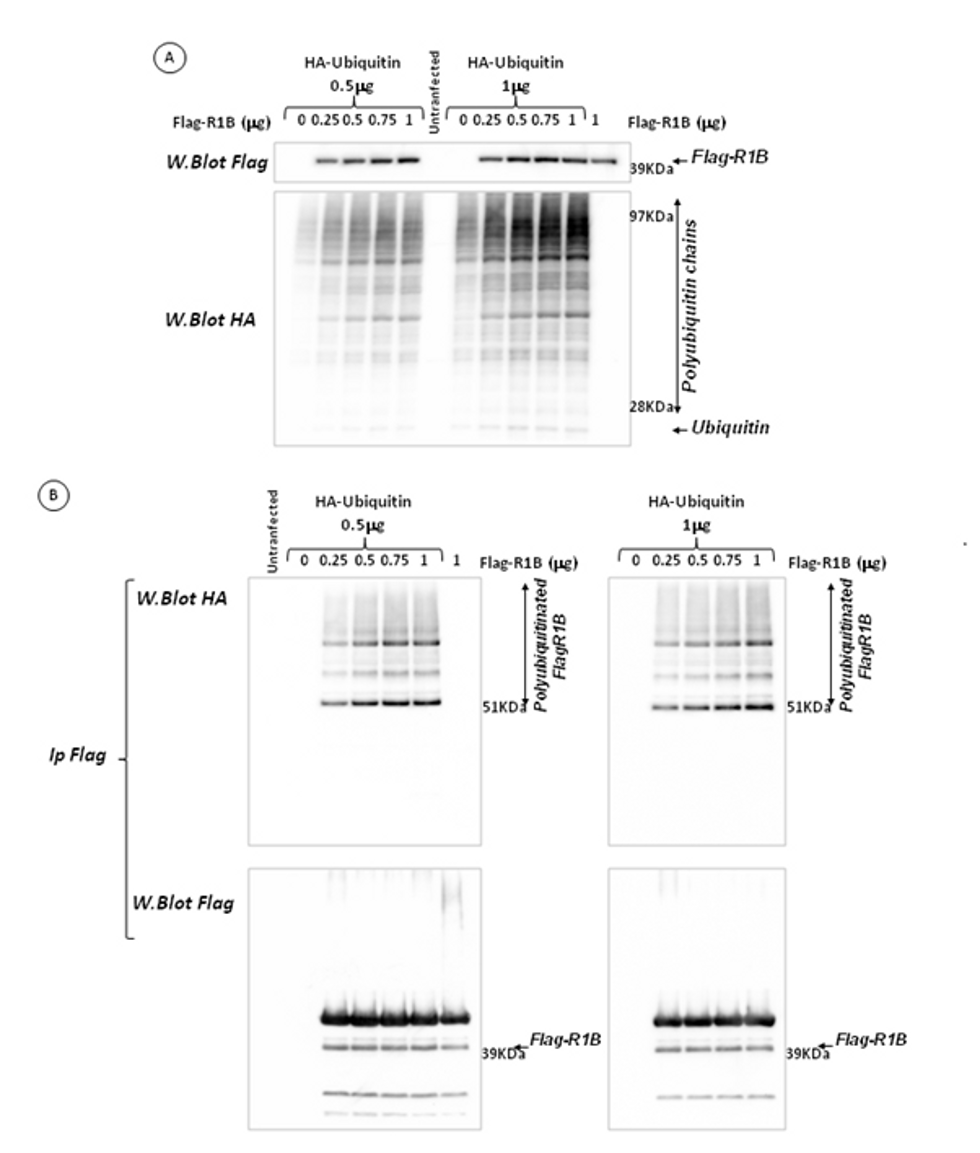

Supplement: Figure S14 — Cellular assay for Ring 1B ubiquitination. Cos-1 cells were co-transfected with the indicated amounts of a FLAG-tagged Ring1B (Flag-R1B) construct and an HA-tagged ubiquitin construct. A. Total lysates were analyzed by Western blotting using antibodies against Flag and HA epitopes. B. Flag immunoprecipitations were performed after normalization of Flag-tagged Ring1B level and were analyzed by Western blotting using antibodies against the HA and Flag epitopes. (TIF) [file pcbi.1002317.s014.tif]
