# Supplementary material for: Switches, Excitable Responses and Oscillations in the Ring1B/Bmi1 Ubiquitination System
Source: PLoS Comput Biol. 2011 Dec 15;7(12):e1002317. doi: 10.1371/journal.pcbi.1002317 (PMC3240587; doi:10.1371/journal.pcbi.1002317)
Supplement: Table S1 — Rate expressions and kinetic parameters of the Michaelis-Menten model. (DOC) [file pcbi.1002317.s015.doc]

**Table S1.** **Rate expressions and kinetic parameters.**

|  | **Reactions** | **Reaction rates** | **Parameters** | **Parameter values** | **Parameter values normalized by [R1B_tot_]** |
| --- | --- | --- | --- | --- | --- |
| *v_1_* | $\text{Bmi1}\to\text{Bmi1}_{\text{ub}}^{\text{d}}$ | $k_{1}\text{[Bmi1]}$ | *k*_1_ | 0.002 (s­^-1^) | 0.002 (s­^-1^) |
| *v_2_* | $\text{Bmi1}^{\text{d}}_{\text{ub}}\to\text{Bmi1}$ | $k_{2}\text{[Bmi1}_{\text{ub}}^{\text{d}}\text{]}$ | *k*_2_ | 0.002 (s­^-1^) | 0.002 (s­^-1^) |
| *v_3_* | $\text{R1B}\to\text{R1B}_{\text{ub}}^{\text{d}}$ | $k_{3}\text{[R1B]}$ | *k*_3_ | 0.01 (s­^-1^) | 0.01 (s­^-1^) |
| *v_4_* | $\text{R1B1}_{\text{ub}}^{\text{d}}\to\text{R1B}$ | $k_{4}\text{[USP7}_{\text{tot}}\text{][R1B}_{\text{ub}}^{\text{d}}\text{]}$ | *k*_4_ | 10^-5^ (nM^-1^ s­^-1^) | 0.001 (s­^-1^) |
| *v_5_* | $\text{Bmi1}+\text{R1B}\leftrightarrow\text{R1B}_{\text{ub}}^{\text{d}}$ | $k_{5}\text{[R1B][Bmi1]}-k_{5r}\text{[Z]}$ | *k*_5_  *k*_5r_ | 0.02 (nM^-1^ s­^-1^)  0.2 (s­^-1^) | 2 (s­^-1^)  0.2 (s­^-1^) |
| ** v_6_* | $\text{Z}\to Z_{\text{ub}}$ | $[Z](k_{6}\text{[Z]}+k_{6a}[\text{Z}_{\text{ub}}])$ | *k*_6_  *k*_6a_ | 2·10^-4^ (nM^-1^ s­^-1^)  0.002 (nM^-1^ s­^-1^) | 0.02 (s­^-1^)  0.2 (s­^-1^) |
| *v_7_* | $Z_{\text{ub}}\to Z$ | $\frac{k_{7}\text{[USP7}_{\text{tot}}\text{][Z}_{\text{ub}}]}{K_{M7}+\text{[Z}_{\text{ub}}\text{]}}$ | *k_7_*  *K*_M7_ | 0.005 (s­^-1^)  0.25 (nM) | 0.005 (s­^-1^)  0.0025 |
| *v_8_* | $\text{Z}_{\text{ub}}\leftrightarrow\text{R1B}_{\text{ub}}^{\text{a}}+\text{Bmi1}$ | $\begin{matrix} k_{8}[\text{Z}_{\text{ub}}\text{]} \\ -k_{8r}\text{[R1B}_{\text{ub}}^{\text{a}}\text{][Bmi1]} \end{matrix}$ | *k*_8_  *k*_8r_ | 0.012 (s­^-1^)  2·10^-7^ (nM^-1^ s­^-1^) | 0.012(s­^-1^)  2·10^-5^ (s­^-1^) |
| ** v_9_* | $\text{R1B}\to\text{R1B}_{\text{ub}}$ | $\begin{matrix} \text{[R1B}](k_{9}\text{[R1B]} \\ +k_{9a}\text{[R1B}_{\text{ub}}\text{])} \end{matrix}$ | *k*_9_  *k*_9a_ | 0.002 (nM^-1^ s­^-1^)  0.002 (nM^-1^ s­^-1^) | 0.2 (s­^-1^)  0.2 (s­^-1^) |
| *v_10_* | $\text{R1B}_{\text{ub}}\to\text{R1B}$ | $k_{10}\text{[USP7}_{\text{tot}}\text{][R1B}_{\text{ub}}\text{]}$ | *k*_10_ | 7.5·10^-5^ (nM^-1^ s­^-1^) | 0.0075 (s­^-1^) |
| *v_11_* | $\text{R1B}_{\text{ub}}^{\text{a}}\to\text{R1B}$ | $k_{11}\text{[USP7}_{\text{tot}}\text{][R1B}_{\text{ub}}^{\text{a}}\text{]}$ | *k*_11_ | 5·10^-5^ (nM^-1^ s­^-1^) | 0.005 (s­^-1^) |
| *v_12_* | $\text{H2A}\to\text{H2A}_{\text{ub}}^{\text{a}}$ |  | *k*_12a_  *k*_12b_  *k*_12c_ | 2·10^-5^ (nM^-1^ s­^-1^)  0.02 (nM^-1^ s­^-1^)  0.002 (nM^-1^ s­^-1^) | 0.002 (s­^-1^)  2 (s­^-1^)  0.2 (s­^-1^) |
| *v_13_* | $\text{H2A}_{\text{ub}}^{\text{a}}\to\text{H2A}$ | $k_{13}\text{[H2A}_{\text{ub}}\text{]}$ | *k*_13_ | 0.01 (s­^-1^) | 0.01 (s­^-1^) |
| $v_{prod}^{R1B}$ | $...\to\text{R1B}$ | $v_{prod}^{R1B}$ | $v_{prod}^{R1B}$ | 7.5·10^-4^ (nM s­^-1^) | 7.5·10^-6^ (s­^-1^) |
| $v_{\deg}^{R1B}$ | $\text{R1B1}_{\text{ub}}^{\text{d}}\to...$ | $k_{\deg}^{R1B}\text{[R1B}_{\text{ub}}^{\text{d}}\text{]}$ | $k_{\deg}^{R1B}$ | 3·10^-5^ (s­^-1^) | 3·10^-5^ (s­^-1^) |
| $v_{prod}^{Bmi1}$ | $...\to\text{Bmi1}$ | $v_{prod}^{Bmi1}$ | $v_{prod}^{Bmi1}$ | 7.5·10^-4^ (nM s­^-1^) | 7.5·10^-6^ (s­^-1^) |
| $v_{\deg}^{Bmi1}$ | $\text{Bmi1}_{\text{ub}}^{\text{d}}\to...$ | $k_{\deg}^{Bmi1}\text{[Bmi1}_{\text{ub}}^{\text{d}}]$ | $k_{\deg}^{Bmi1}$ | 3·10^-5^ (s­^-1^) | 3·10^-5^ (s­^-1^) |
|  |  |  | [R1B_tot_] | 100 nM | 1 |
|  |  |  | [Bmi1_tot_] | 100 nM | 1 |
|  |  |  | [H2A_tot_] | 10 nM | 0.1 |
|  |  |  | [USP7_tot_] | 100 nM | 1 |

*See supplementary section 3: “Derivation of Auto-ubiquitination reaction Rates”.
