# Supplementary material for: Switches, Excitable Responses and Oscillations in the Ring1B/Bmi1 Ubiquitination System
Source: PLoS Comput Biol. 2011 Dec 15;7(12):e1002317. doi: 10.1371/journal.pcbi.1002317 (PMC3240587; doi:10.1371/journal.pcbi.1002317)
Supplement: Text S1 — Derivation of the Michaelis-Menten model and Mass-Action model describing the dynamics of the Ring1B/Bmi1 ubiquitination system and comparison of model predictions (sections 1–3 and 5). Theoretical background on Linear Stability Analysis (section 4) and Preliminary Experimental Validation of Model Predictions (section 6). (DOC) [file pcbi.1002317.s017.doc]

**Supporting Information - Text S1**

**Content:**

1. System of Equations Describing the Dynamics of the Ring1B/Bmi1 Ubiquitination System

2. Rate Equations and Parameter Values of the Ring1B/Bmi1 System Used in Model Analysis.

3. Derivation of Auto-ubiquitination Reaction Rates

4. Linear Stability Analysis

5. Comparison of an Approximate Michaelis-Menten Description and an Elementary Step Description (a Mass-Action Model).

6. Preliminary Experimental Validation of Model Predictions

**1. System of Equations Describing the Dynamics of the Ring1B/Bmi1 Ubiquitination System**

The MM model used in the main text is described by the following ODEs^*^:

$\frac{d[Bmi1^{d}_{ub}]}{dt}=v_{1}-v_{2}-v_{\deg}^{Bmi1}$, (S1)

$\frac{d[Bmi1]}{dt}=-v_{1}+v_{2}-v_{5}+v_{8}+v_{prod}^{Bmi1}$, (S2)

$\frac{d[R1B^{d}_{ub}]}{dt}=v_{3}-v_{4}-v_{\deg}^{R1B}$, (S3)

$\frac{d[R1B]}{dt}=-v_{3}+v_{4}-v_{5}-v_{9}+v_{10}+v_{11}+v_{prod}^{R1B}$, (S4)

$\frac{d[Z]}{dt}=v_{5}-v_{6}+v_{7}$, (S5)

$\frac{d[Z_{ub}]}{dt}=v_{6}-v_{7}-v_{8}$, (S6)

$\frac{d[R1B^{a}_{ub}]}{dt}=v_{8}-v_{11}$, (S7)

$\frac{d[R1B_{ub}]}{dt}=v_{9}-v_{10}$, (S8)

$\frac{d[H2A_{ub}]}{dt}=v_{12}-v_{13}$, (S9)

$\frac{d[H2A]}{dt}=-v_{12}+v_{13},$, (S10)

^*^The rate expressions are given in Table S1 below. For a short-timescale model that is analyzed in the main text, the protein synthesis and degradation rates ($v_{prod}^{R1B}$,$v_{\deg}^{R1B}$,$v_{prod}^{Bmi1}$,$v_{\deg}^{Bmi1}$- Table S1) are neglected. For a long-timescale model the degradation of the complex Z is neglected, because the experimental data suggest that its half-life is about three times larger (~7 - 8 hrs) than the half-lives of free Ring1B and Bmi1, which are about 3 hrs [1]. Inclusion of Z degradation into a model does not appreciably change the complex dynamics observed over the first hour of the response.

**2. Rate Equations and Parameter Values of the Ring1B/Bmi1 System Used in Model Analysis.**

The lack of experimentally measured kinetic data remains a challenge for model-based studies. For both the short- and long-timescale models, the kinetic parameters are constrained by experimental data (wherever available) or typical values for protein association/dissociation and enzymatic reaction rates. In the long-timescale model, we use 3·10^-5^ (s^-1^) for the rate constants of Ring1B and Bmi1 degradation, which is comparable to the experimental data on the half-lives of free Ring1B and Bmi1 [1]. See Table S1.

**3. Derivation of Auto-ubiquitination Reaction Rates**
 A critical nonlinearity of the system is brought about by the intermolecular auto-ubiquitination of Ring1B when free and/or when in complex with Bmi1. Here, we give derivation of the form of the reaction rates *v*_6_ and *v*_9_ used in our model which characterize the auto-ubiquitination steps of the Ring1B-Bmi1 complex (Z) and free Ring1B (R1B).
 Because both Z and Z _ub_ can catalyse the auto-ubiquitination of Z, the following processes are involved in this step:


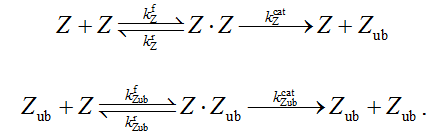


The auto-ubiquitination rate *v*_6_ is the sum of the rates catalysed by each form, Z and Z_ub_. Applying quasi steady-state (QSS) approximation for the intermediate complexes, we obtain the following expression for *v*_6_:

$$v_{6}=[Z]\left(\frac{k_{\text{Z}}^{\text{cat}}}{K_{\text{Z}}}[Z]+\frac{k_{\text{Zub}}^{\text{cat}}}{K_{\text{Zub}}}[Z_{ub}]\right)$$

where $k_{\text{Z}}^{\text{cat}}$, $k_{\text{Zub}}^{\text{cat}}$and $K_{\text{Z}}=(k_{\text{Z}}^{\text{r}}+k_{\text{Z}}^{\text{cat}})/k_{\text{Z}}^{\text{f}}$, $K_{\text{Zub}}=(k_{\text{Zub}}^{\text{r}}+k_{\text{Zub}}^{\text{cat}})/k_{\text{Zub}}^{\text{f}}$are the catalytic and Michaelis constants, respectively, of component processes involved in the above reactions. Let $k_{6}=\frac{k_{\text{Z}}^{\text{cat}}}{K_{\text{Z}}},k_{6\text{a}}=\frac{k_{\text{Zub}}^{\text{cat}}}{K_{\text{Zub}}}$, *v*_6_ becomes $v_{6}=[Z]\left(k_{6}[Z]+k_{6\text{a}}[Z_{ub}]\right)$.

The form of *v*_9_ can be obtained in a similar way via the following reactions:


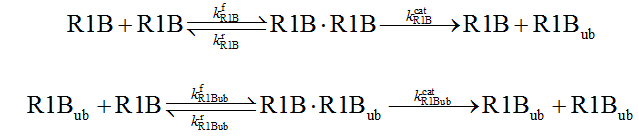


Again, applying QSS approximation for the intermediate complexes, we obtain:

$v_{9}=[\text{R1B}]\left(k_{9}[\text{R1B}]+k_{9\text{a}}[\text{R1B}_{ub}]\right)$,

with $k_{9}=\frac{k_{\text{R1B}}^{\text{cat}}}{K_{\text{R1B}}},k_{9\text{a}}=\frac{k_{\text{R1Bub}}^{\text{cat}}}{K_{\text{R1Bub}}}$where $K_{\text{R1B}}=(k_{\text{R1B}}^{\text{r}}+k_{\text{R1B}}^{\text{cat}})/k_{\text{R1B}}^{\text{f}}$and $K_{\text{R1Bub}}=(k_{\text{R1Bub}}^{\text{r}}+k_{\text{R1Bub}}^{\text{cat}})/k_{\text{R1Bub}}^{\text{f}}$.

**4. Linear Stability Analysis** Tools of nonlinear dynamics provide a useful framework to examine the dynamical properties of the Ring1B/Bmi1 system described by equations S1-10. We are mainly interested in the asymptotic states and transitions between different dynamic regimes. The steady states of the system are obtained by equating equations S1-10 to zero and solving for the values of the concentrations. In the case that synthesis and degradation are neglected, there are seven independent equations and three additional conditions are required to solve the system. |These conditions are the total mass conservation for the R1B, Bmi1, and H2A species which are written as

$[Bmi1_{tot}]=[Bmi1^{d}_{ub}]+[Bmi1]+[Z]+[Z_{ub}]$, (S11)

$[R1B_{tot}]=[R1B^{d}_{ub}]+[R1B]+[Z]+[Z_{ub}]+[R1B^{a}_{ub}]+[R1B_{ub}]$, (S12)

$[H2A_{tot}]=[H2A_{ub}]+[H2A]$. (S13)

In this case, a solution different from the trivial one (all the species equal to zero) can exist and the temporal evolution of the system can be described by only seven independent selected species. If we assume that ***S***_indep_ is the vector composed by these independent concentrations, the temporal evolution of the systems is completely determined by

$\frac{dS_{\text{indep}}}{dt}=f(S_{\text{indep}}),$ (S14)

where *f*(***S***_indep_) is the vector formed by the reaction rates of the independent selected species. The steady states denoted by ***S***^0^_indep_ are obtained by solving *f*(***S***^0^_indep_)=0. The asymptotic stability of these steady states can be determined by linear analysis upon perturbations. Thus, in the vicinity of any of these steady states the temporal evolution of a perturbation from this state, denoted by Δ***S***^0^_indep_ =***S***_indep_ ***-S***^0^_indep_, is given by

$\frac{d\Delta S_{\text{indep}}}{dt}=J(S_{\text{indep}})\Delta S_{\text{indep}}^{0},$ (S15)

where *J*(***S***^0^_indep_) is the Jacobian matrix of the reduced system evaluated at the considered steady state. Thus, the dynamical behavior of the system is entirely specified by the Jacobian matrix and by its eigenvalues and eigenvectors. If we assume that the eigenvalues of *J*(***S***^0^_indep_) for a given steady state ***S***^0^_indep_, denoted by λ_i_ (i=1,2,..7) are ordered decreasingly by the values of their real part, Re(λ_i_), then the dominant growth term of the perturbation is governed by

$\Delta S_{\text{indep}}^{0}(t)\propto E_{1}\text{e}^{\text{Re}(\lambda_{1})t},$ (S16)

where ***E***_1_ is the eigenvector corresponding to the eigenvalue with the largest real part. The state ***S***^0^_indep_ is asymptotically stable if, and only if, Re(λ_1_) is negative and Δ***S***^0^_indep_ tends to exponentially decrease in the course of time. If there is at least one positive eigenvalues’ real part (i.e. at least Re(λ_i_) is positive) then the perturbation grows exponentially and ***S***^0^_indep_ is unstable in response to the perturbation.

In order to study the asymptotic states and the transition between different dynamical behaviors, we have solved numerically the system for the parameters values given in table S1 (and the rest of values mentioned in the text) using Maple^TM^ 13.0. A useful step in obtaining the steady states is to reduce the system of seven independent variables to a less nonlinear system with a smaller number of variables. Equating Eq. S7 to zero and expressing [R1B] as a function of [R1B_ub_] as

$[R1B]=\frac{-k_{9a}[R1B_{ub}]+\sqrt{(k_{9a}[R1B_{ub}])^{2}+4k_{9}k_{10}[R1B_{ub}]}}{2k_{9}}$, (S17)

since the other solution of the quadratic equation is always negative.
 For the proposed Ring1B/Bmi1 model and the considered parameter ranges, only one or three real positive solutions are possible. Once the solutions have been obtained, we have substituted them into the Jacobian Matrix and numerically calculated their eigenvalues to classify the different dynamical behaviors. As shown in Fig. 4 we have repeated this procedure for different values of the normalized Bmi1 and USP7 abundance to obtain a more complete picture of the system dynamics on the parameter space.
 When a unique steady state exists, the sign of the largest real part of the eigenvalue associated with that solution allows us to classify that steady state as stable (represented by cyan in Fig. 4), or unstable (green in Fig. 4) indicating the presence of sustained oscillations. The same analysis is possible when three steady states coexist. Evaluating the Jacobian matrix and calculating the eigenvalues for each solution enabled classification of the possible dynamical behaviors. For the considered ranges of parameters, two different cases were reported: (1) two stable and one unstable solution indicating a bistable dynamical behavior (represented by the red in Fig. 4), and (2) one stable and two unstable solutions suggesting a possible excitable behavior (yellow in Fig. 4). The borders separating these different regions correspond to different kind of transitions. The points belonging to the borders separating the cases when one or three solutions exist (i.e. the lines separating the blue and green regions from the red and yellow regions) are called saddle nodes. The points belonging to the borders where any of the steady states lose its stability (the largest negative real part of the eigenvalue becomes positive) as in the transitions between the blue and green regions and between the red and yellow regions, are called Hopf bifurcations.

**5. Comparison of an Approximate Michaelis-Menten Description and an Elementary Step Description (a Mass-Action Model).**
 This section presents in detail the development and simulation results of a mass-action (MA) model (Fig.S9) for the Ring1B/Bmi1/H2A system in comparison with a Michaelis-Menten (MM) model. We show that the complex dynamics (bistability, oscillations and excitability) found for the MM model and presented in the main text are also observed for the MA model. Importantly, in the MA model these observed complex dynamics persist for two different cases (a) when the system is within the MM limit (a MM description applies) and (2) when a MM description is inapplicable. This means the intricate dynamic behaviors are inherent properties of the system, which are independent of a modelling framework used.

***5.1 Model ODEs and Parameter values***
 In the MM model, we assumed MM kinetics for the deubiquitination step that converts Z_ub_ into its unubiquitinated form, Z (reaction 7, Fig.1, main text). This step is replaced by two elementary reactions 7 (enzyme-substrate binding) and 7’ (catalytic conversion) in the new MA model (Fig.S9). The MA model includes the new variables, Z_ub_USP7 and USP7, and is described by the following ODEs:

$\frac{d[Bmi1^{d}_{ub}]}{dt}=v_{1}-v_{2}-v_{\deg}^{Bmi1}$, (S18)

$\frac{d[Bmi1]}{dt}=-v_{1}+v_{2}-v_{5}+v_{8}+v_{prod}^{Bmi1}$, (S19)

$\frac{d[R1B^{d}_{ub}]}{dt}=v_{3}-v_{4}-v_{\deg}^{R1B}$, (S20)

$\frac{d[R1B]}{dt}=-v_{3}+v_{4}-v_{5}-v_{9}+v_{10}+v_{11}+v_{prod}^{R1B}$, (S21)

$\frac{d[Z]}{dt}=v_{5}-v_{6}+v_{7'}$, (S22)

$\frac{d[Z_{ub}USP7]}{dt}=v_{7}-v_{7'},$ (S23)

$\frac{d[USP7]}{dt}=v_{7'}-v_{7},$ (S24)

$\frac{d[Z_{ub}]}{dt}=v_{6}-v_{7}-v_{8}$, (S25)

$\frac{d[R1B^{a}_{ub}]}{dt}=v_{8}-v_{11}$, (S26)

$\frac{d[R1B_{ub}]}{dt}=v_{9}-v_{10}$, (S27)

$\frac{d[H2A_{ub}]}{dt}=v_{12}-v_{13}$, (S28)

$\frac{d[H2A]}{dt}=-v_{12}+v_{13},$, (S29)

**5.1.1 Bistability in the MA model** We showed in the main text that bistability in the Ring1B/Bmi1 system arises from positive feedback loops brought about by intermolecular self-induced ubiquitination of Ring1B combined with the saturable Michaelis-Menten (MM) kinetics. However, the MM kinetics may not be applicable when the concentration of USP7 (an enzyme in deubiquitination reaction) is comparable with the Z_ub_ concentration (a substrate). Here we show that the Ring1B/Bmi1 system still exhibits bistability under the condition when saturable MM kinetics cannot be applied.

In this case, to describe the systems stationary behavior, including bistability, one has to use a total quasi-steady state (TQSS) approximation (see in the main text and Refs. [38,39] therein) or consider a detailed elementary step model. The algebraic equations, which determine the steady states within both the TQSS approximation and detailed MA description frameworks, are equivalent to those for MM kinetic equations, but explicitly account for the concentration of the Z_ub_USP7 complex. Clearly, this additional term has little effect when the USP7 abundance is much less than the Ring1B abundance, but can significantly modify the stationary solutions when both abundances are comparable. Although the MM assumption is invalid when the USP7 abundance is comparable to that of the Ring1B, the bistable behavior is observed for the Ring1B/Bmi1 system. Fig.S10a shows bistability for levels of active Ring1B (Z_ub_ and R1B^a^_ub_) in the MA model when the USP7 abundance is high. Moreover, we also observe bistability for level of monoubiquitinated H2A in response to increasing Bmi1 abundance (Fig.S10b). These computational results demonstrate that bistability persists for both detailed and reduced models, suggesting that this dynamics is a robust systems property.

**5.1.2 Oscillations and Excitable Responses in the MA model** Similar to the bistability case, we studied oscillations behavior in the MA model for two scenarios: (1) when the MM assumptions are satisfied, and (2) when the MM assumptions are inapplicable. The deubiquitinise USP7 rate can be described by a MM expression when the abundance of the deubiquitinase USP7 is very small (1 nM) compared to the Ring1B and Bmi1 abundances (>100 nM). In this case, choosing the elementary parameters *k*_7f_, *k*_7r_ and *k*_7cat_ such that the ratio (*k*_7r_ + *k*_7cat_)/ *k*_7f_ is comparable to the *K*_m_ value in the MM model, oscillations are observed in the MA model (Fig. S11a). More importantly, we explore the MA model when the MM assumption of excess substrate relative to enzyme concentration is no longer valid. We found that the system still displays sustained oscillations even when the USP7 abundance is high (100nM), which is comparable to that of Ring1B (Fig.S11b). Varying *k*_7f_ can markedly change the oscillations frequency, suggesting the pattern of oscillations is robust and can be tuned if needed (Fig.S11c). Combined together, these results suggest that the Ring1B/Bmi1/H2A system can display robust oscillatory behavior independently of whether the MM kinetics is applicable or not. Close to the oscillatory regime, excitable, overshoot responses can also be observed (Fig.S11d).

**6. Preliminary Experimental Validation of Model Predictions**
 To test the validity of our computational model, preliminary experiments have been conducted in which we measured steady-state levels of ubiquitinated Ring1B in response to increasing the total Ring1B abundance. To this end, Cos-1 cells were co-transfected with HA--tagged ubiquitin (0.5 µg or 1 µg) and increasing amounts of Flag-tagged Ring1B (0.25, 0.5, 0.75 or 1 µg) (Fig.S14). For each transfection combination, Ring1B was then pulled down using immunoprecipitation against the Flag-tag and checked for ubiquitination status with antibody against the HA-tag. Data quantification in Fig.S12 shows, at both levels of transfected ubiquitin, a linear correlation between the total of all ubiquitinated Ring1B forms and the total abundance of Ring1B. The measured ubiquitinated Ring1B will be in a range between the total active Ring1B (R1B_ub_ + Z_ub_ + R1B^a^_ub_) and the total ubiquitinated Ring1B (R1B_ub_ + Z_ub_ + R1B^a^_ub_ + R1B­^d^_ub_); note that since Ring1B degradation is not explicitly considered in a short-term model, the amount of R1B^d^_ub_ is slightly overestimated. Fig.S13 shows model predictions of the total active Ring1B and the total uqibiquitinated Ring1B versus the total Ring1B abundance. In both cases, consistent with the observed experimental data, the model predicts a linear relationship between the ubiquitinated Ring1B concentrations and the total Ring1B, particularly for Ring1B abundance larger than 100 nM. Moreover, parameter sensitivity analysis suggested that such relationship is quite robust against parameter variations. Despite a bistable response is exhibited, the bistability range and the lower steady-state to higher steady-state jump are negligible such that potential switching is unlikely to be detected by experiments. Although these experiments are not decisive in confirming if the predicted dynamics presented in the manuscript (switches, oscillations or excitable response) are realized in the tested cells, they provide convincing experimental support for our model validity.
 To verify other model predictions, we have been conducting a number of additional experiments. To detect bistable response or hysteresis we increasingly over-express GST-tagged USP7 co-transfected with HA-ubiquitin and Flag-Ring1B to measure the abundances of the different ubiquitinated Ring1B forms. In addition, we knockdown USP7 using increasing amounts of siRNA, then co-transfecting HA-ubiquitin and Flag-Ring1B and measure the ubiquitinated Ring1B levels. A similar setup involves varying the Bmi1 abundance instead of USP7. Although the results are interesting, these experiments have proved rather challenging to be conclusive. This is due to several reasons. Most importantly, lack of linkage specific antibodies makes it currently impossible to differentiate between ubiquitinated Ring1B forms characterized by differently ubiquitin linkages, let alone Ring1B that are modified by mixed linkages which typically are the case in the Ring1B/Bmi1 system. This technical limitation prevents current effort to separately measure Z_ub_, R1B_ub_ and R1B­^d^_ub_. In reality, the ubiquitinated Ring1B measured in our experiments encompasses all forms of ubiquitin-modified Ring1B, whereas observables such as Z_ub_ or R1B_ub_ are the key in verifying model prediction.
 Because of these experimental limitations, the more complete verification of model predictions requires time and effort that goes beyond the scope of the current paper. As this is the first mathematical model of the Ring1B/Bmi1 ubiquitination system, our main objective is to draw attention to a rich repertoire of dynamical behaviors that the system can exhibit, which hopefully stimulates exciting follow-up experiments by other groups.

**Experimental Procedure: *Cell Culture and Transfection.*** Cos-1 cells were maintained in DMEM medium with 10% fetal bovine serum (Invitrogen/GIBCO). Transfection of *Cos-1* cells was performed using Lipofectamine 2000 (Invitrogen). Plasmids encoding Flag-R1B and HA-Ubiquitin were used in transfection as indicated***.
 Cellular ubiquitin assay.*** 48hours after transfection cells were washed with ice-cold phosphate-buffered saline and lysed in lysis buffer containing 50mM tris-HCl pH 7.4, 150mM NaCl, 1% nonidet, 0.25% sodium deoxycholate, 1mM EDTA, 1mM EGTA, 50mM sodium fluoride, 10mM β-glycerophosphate, 5mM sodium pyrophosphate, 0.2mM phenylmethylsulfonyl fluoride, 1mM benzamidine, 10mg/ml aprotinin, and 10mg/ml leupeptin (Sigma).
 Flag-Ring1B was immunoprecipitated using anti–Flag antibody M2 agarose conjugated (Sigma). The samples were resolved by SDS-PAGE, followed by immunoblot analysis using anti-FLAG antibody M2- HRP (Sigma) or anti–HA-HRP3F10 (Roche).

**References**

1. Ben-Saadon R, Zaaroor D, Ziv T, Ciechanover A (2006) The polycomb protein Ring1B generates self atypical mixed ubiquitin chains required for its in vitro histone H2A ligase activity. Mol Cell 24: 701-711.
